# Genetic and physiological characterization of sunflower resistance provided by the wild-derived *Or*_*Deb2*_ gene against highly virulent races of *Orobanche cumana* Wallr

**DOI:** 10.1007/s00122-021-03979-9

**Published:** 2021-11-06

**Authors:** Mónica Fernández-Aparicio, Lidia del Moral, Stéphane Muños, Leonardo Velasco, Begoña Pérez-Vich

**Affiliations:** 1 Instituto de Agricultura Sostenible (IAS-CSIC), Alameda del Obispo s/n, 14004 Córdoba, Spain; 2 Laboratoire des Interactions Plantes Microbes-Environnement (LIPME), CNRS, INRAE, Université de Toulouse, Castanet-Tolosan, France

## Abstract

**Key message:**

*Or*_*Deb2*_ confers post-attachment resistance to *Orobanche cumana* and is located in a 1.38 Mbp genomic interval containing a cluster of receptor-like kinase and receptor-like protein genes with nine high-confidence candidates.

**Abstract:**

Sunflower broomrape is a holoparasitic angiosperm that parasitizes on sunflower roots, severely constraining crop yield. Breeding for resistance is the most effective method of control. *Or*_*Deb2*_ is a dominant resistance gene introgressed into cultivated sunflower from a wild-related species that confers resistance to highly virulent broomrape races. The objectives of this study were as follows: (i) locate *Or*_*Deb2*_ into the sunflower genome and determine putative candidate genes and (ii) characterize its underlying resistance mechanism. A segregating population from a cross between the sunflower resistant line DEB2, carrying *Or*_*Deb2*_, and a susceptible line was phenotyped for broomrape resistance in four experiments, including different environments and two broomrape races (F_GV_ and G_TK_). This population was also densely genotyped with microsatellite and SNP markers, which allowed locating *Or*_*Deb2*_ within a 0.9 cM interval in the upper half of Chromosome 4. This interval corresponded to a 1.38 Mbp genomic region of the sunflower reference genome that contained a cluster of genes encoding LRR (leucine-rich repeat) receptor-like proteins lacking a cytoplasmic kinase domain and receptor-like kinases with one or two kinase domains and lacking an extracellular LRR region, which were valuable candidates for *Or*_*Deb2*_. Rhizotron and histological studies showed that *Or*_*Deb2*_ determines a post-attachment resistance response that blocks *O. cumana* development mainly at the cortex before the establishment of host-parasite vascular connections. This study will contribute to understand the interaction between crops and parasitic weeds, to establish durable breeding strategies based on genetic resistance and provide useful tools for marker-assisted selection and *Or*_*Deb2*_ map-based cloning.

**Supplementary Information:**

The online version contains supplementary material available at 10.1007/s00122-021-03979-9.

## Introduction

Sunflower broomrape (*Orobanche cumana* Wallr.) is a holoparasitic plant that parasitizes the roots of sunflower (*Helianthus annuus* L.) and constraints its production in large areas of the Old World. The species has been reported to occur in most of the sunflower producing regions of Spain and France, around the Black and Caspian Seas, in China, and, recently, in some parts of Africa (Fernández-Martínez et al. 2015). The use of resistant sunflower cultivars is one of the most efficient control methods for this parasitic weed, and it has been widely used since early sunflower breeding in the former USSR to present times (Fernández-Martínez et al. 2015). Unlike other *Orobanche* and *Phelipanche* weedy species, for which genetic resistance in the host is of quantitative nature (horizontal), genetic resistance to *O. cumana* in sunflower is in most cases qualitative or vertical and controlled by major dominant genes (Pérez-Vich et al. 2013). For this reason, *O. cumana* populations are commonly classified into physiological races (Vranceanu et al. 1980). The genetic control of broomrape resistance by a single dominant gene was first reported by Pogorletsky and Geshele (1976). Shortly after, Vranceanu et al. (1980) identified five *O. cumana* races named A to E which were controlled by five dominant resistant genes named *Or1* to *Or5*, respectively. Several studies confirmed monogenic dominant resistance to race E (Sukno et al. 1999; Lu et al. 2000; Pérez-Vich et al. 2004). Similarly, one dominant gene was reported controlling races which overcome *Or5* resistance. Pacureanu-Joita et al. (2004) described *Or6* present in the line LC-1093 as conferring resistance to Romanian race F broomrape populations, and Duriez et al. (2019) reported *HaOr7* controlling Spanish race F populations, although the *HaOr7* gene has also been reported as conferring resistance to *O. cumana* populations more virulent than those classified as race F (Martín-Sanz et al. 2020). For race G, Velasco et al. (2012) detailed that resistance transferred from *H. debilis* subsp. *tardiflorus* was determined also by a single dominant gene, *Or*_*Deb2*_. Finally, a sunflower post-vascular connection resistance to *O. cumana* races F and G named as “System II resistance” has been reported to be controlled by the single partially dominant gene *Or*_*SII*_ (Hassan et al. 2008; Martín-Sanz et al. 2020). In addition to this major gene effect, a quantitative component of broomrape resistance determined by quantitative trait loci (QTLs) that contribute with small-to-moderate effects to decrease the number of emerged broomrapes has also been described (Pérez-Vich et al. 2004; Akhtouch et al. 2016; Louarn et al. 2016; Imerovski et al. 2019).

A number of studies for mapping the major broomrape resistance genes have been reported. The first gene mapped was *Or5*, which was located on a telomeric region of chromosome (Chr) 3 of the sunflower genetic map (Lu et al. 2000; Tang et al. 2003; Pérez-Vich et al. 2004). Imerovski et al. (2013) also found simple sequence repeat (SSR) markers of Chr3 strongly associated with resistance genes other than *Or5* such as *Or2*, *Or4*, and *Or6*, conferring resistance to races B, D, and F, respectively. Also on Chr3, Imerovski et al. (2016, 2019) mapped the recessive gene *orab-vl-8* that provides resistance to races higher than F and a major QTL of recessive nature determining resistance to race G, both located in an *Or5* non-overlapping region. Other major resistance genes have been reported to map on chromosomes other than Chr3. Duriez et al. (2019) have mapped the *HaOr7* gene to Chr7, and Hassan et al. (2008) and Martín-Sanz et al. (2020) have located *Or*_*SII*_ to the upper half of Chr4. Recently, the *Or*_*Deb2*_ gene conferring resistance to race G has also been shown to map on Chr4 in a patent application between proprietary SNP markers DHAI000240 and DHAI007796 (Gao et al. 2018).

In the broomrape-host crop interaction, various resistance mechanisms can operate at the pre-attachment or post-attachment stages according to whether the resistance occurs before or after the haustorium attaches the host root surface (Scholes and Press 2008; Timko and Scholes 2013). At the pre-attachment level, the main resistance mechanisms consist of low exudation of germination stimulants, exudation of germination inhibitors, and exudation of inhibitors of radicle development (Höniges et al. 2008). In sunflower, resistant germplasm with low exudation of germination stimulants (line LR1; Labrousse et al. 2001) and with exudation of 7-hydroxylated simple coumarins, which inhibit *O. cumana* seed germination (cultivar Cortés; Serghini et al. 2001), has been reported. Post-attachment mechanisms operate in a first step between the initial contact with the host root and the establishment of effective vascular connections (Pérez-de-Luque et al. 2009). Several different mechanisms consisting of the development of physical barriers such as lignification, suberization, protein crosslinking, or callose accumulation that impede penetration of the parasitic invasive structures, and the production of chemical compounds such as phenolics that are toxic to the parasite, have been described in sunflower (Echevarria-Zomeño et al. 2006; Letousey et al. 2007). Some post-attachment mechanisms operate in a second step after the haustorium has established vascular connections and tubercles are visible (Pérez-de-Luque et al. 2009). Necrosis and subsequent death of broomrape tubercles at several stages of development have been reported in sunflower line LR1 (Labrousse et al. 2001; Louarn et al. 2016). In sunflower line PHSC1102-O carrying the *Or*_*SII*_ resistance gene, phenolic compounds are involved in the delayed parasite development also after host-parasite vascular connections have been established (Martín-Sanz et al. 2020).

Although the major *Or*_*Deb2*_ gene has been located on Chr4 of the sunflower genetic map in a patent application (Gao et al. 2018), detailed information regarding its genetic mapping and the resistance mechanisms associated has not been reported. Consequently, the objectives of the present research were to: (i) confirm Chr4 location of *Or*_*Deb2*_ through a SNP-based bulked segregant analysis, (ii) develop a linkage map including *Or*_*Deb2*_ using publicly available SSR, SNP, and resistance candidate gene markers, (iii) identify candidate genes underlying *Or*_*Deb2*_, and (iv) characterize the *Or*_*Deb2*_-associated physiological resistance mechanisms.

## Materials and methods

### Plant materials

#### Sunflower material

The DEB2 line containing the *Or*_*Deb2*_ major resistance gene (Velasco et al. 2012) and the susceptible line IAS-31 were used for the genetic study. DEB2 was developed by Velasco et al. (2012) through interspecific hybridization with an accession of *Helianthus debilis* subsp. *tardiflorus* and possesses resistance to a broad spectrum of *O. cumana* populations, including populations from races: (i) E from the Guadalquivir Valley (GV) (Southern Spain) (named as E_GV_), F from GV (named F_GV_), (iii) G from GV (named G_GV_), and (iv) G from Eastern European countries (including G from Turkey, named G_TK_) (Velasco et al. 2012; Martín-Sanz et al. 2016). Genetic resistance to *O. cumana* in this line is conferred by dominant alleles at a single gene (Velasco et al. 2012) named as *Or*_*Deb2*_ and reported in a patent application to be located at linkage group 4 of the genetic map of sunflower (Gao et al. 2018). Line IAS-31 is a confectionery inbred line available at IAS-CSIC germplasm collection. It was used in this study because it is very susceptible to all tested *O. cumana* populations of current (F_GV_, G_GV_, G_TK_) and former (E_GV_) races, which indicates that it does not possess major resistance genes.

For the physiological characterization of resistance mechanisms, the control sunflower lines B117, NR5, and P96, in addition to DEB2, were also used. B117 is a confectionery inbred line susceptible to all tested *O. cumana* races (Martín-Sanz et al. 2016). NR5 is an inbred line resistant to E_GV_ and susceptible F_GV_ (Martín-Sanz et al. 2016). P96 is an inbred line resistant to races F_GV_ and G_GV_, and susceptible to races G from Eastern European countries (including G_TK_) (Martín-Sanz et al. 2016).

### Orobanche cumana *populations*

Sunflower broomrape (*Orobanche cumana* Wallr.) populations used for the physiological and the genetic studies were as follows: GT, collected in Turkey and classified as race G_TK_, and SP, collected in Guadalquivir Valley (GV) (Southern Spain) and classified as race F_GV._ The GT broomrape population is classified as race G_TK_ because it parasitized on plants of race F sunflower resistant lines K-96, P96 and R-96 (Fernández-Martínez et al. 2004) and race F resistant sunflower population BR4 (Jan et al. 2002; Velasco et al. 2012). The SP population is classified as race F_GV_ because it parasitized on plants of the race E resistant lines NR5 and P-1380 and hybrid P64LE19 and did not parasitize plants of the race F resistant line P96 (Rodríguez-Ojeda et al. 2013; Martín-Sanz et al. 2016). In addition to populations GT and SP, the OC-94 *O. cumana* population also collected in the Guadalquivir Valley of Spain and classified as race E_GV_ was also used for the *Or*_*Deb2*_ physiological characterization. Population OC-94 is classified as race E_GV_ because it shows virulence on plants of cultivar S-1358, which is resistant to races A through D and susceptible to race E, and avirulence on plants of cultivar P-1380, carrying the *Or5* gene that confers resistance to race E (Rodríguez-Ojeda et al. 2013).

### Genetic mapping study

#### Mapping population and phenotyping

Immature florets of IAS-31 plants were emasculated and pollinated with pollen of DEB2 plants. An F_2_ population consisting of 278 F_2_ plants from an F_1_ self-pollinated plant was obtained. All F_2_ plants were self-pollinated, and F_3_ seeds (i.e., F_2:3_ families) were obtained. For genetic mapping of the *Or*_*Deb2*_ gene, only F_2_ plants for which there was sufficient F_3_ seed for phenotyping their corresponding F_2:3_ families were used; accordingly, the mapping population consisted in 232 F_2_ genotypes for which the F_3_ plant generation could be evaluated. Phenotypic evaluations for broomrape resistance were carried out using the facilities available at the Institute for Sustainable Agriculture (IAS-CSIC, Córdoba, Spain) in four different assays detailed as follows and summarized in Table 1:

- All the 278 F_2_ plants were evaluated for resistance to race G_TK_ in pots under open air conditions. From these 278 F_2_ plants, a subset of 232 with available F_3_ seed constituted the mapping population.
- All the 232 F_2:3_ families from the mapping population were evaluated again for resistance to race G_TK_ in pots under open air conditions. Evaluations were based on 10–12 plants per each F_2:3_ family.
- A total of 220 F_2:3_ families from the mapping population (due to seed availability) were also evaluated for resistance to race F_GV_ in the field. Evaluations were based on 10–12 plants per each F_2:3_ family.
- All F_2:3_ families scored as resistant non-segregating (as described below) after the field and pots evaluations were further confirmed for their reaction to the G_TK_ broomrape race in multi-pot tray assays (Rodríguez-Ojeda et al. 2013). Fifteen plants were evaluated for each F_2:3_ family.

**Table 1:** Phenotyping details for the IAS-31 × DEB2 mapping population

Generation tested	Number of individual F_2_ plants evaluated	Number of F_2:3_ families evaluated (number of F_3_ plants per family)	Broomrape race	Type of assay
F_2_	278 F_2_ plants		G_TK_	Pots
F_3_		232 F_2:3_ families (10–12 F_3_ plants)	G_TK_	Pots
F_3_		220 F_2:3_ families (10–12 F_3_ plants)	F_GV_	Field
F_3_		51 F_2:3_ families (15 F_3_ plants)	G_TK_	Multi-pot tray

In all experiments, the resistant DEB2 and the susceptible IAS-31 parental lines were used as controls.

#### Plant growth conditions and phenotyping for the pot assays

For the pots assays, sunflower seeds were germinated in moistened filter paper and sown in small pots (7 × 7 × 7 cm) containing a mixture of sand and peat (1:1, by vol.) together with 50 mg of broomrape seeds of the GT population (race G_TK_). The plants were kept in a growth chamber for 20–25 days for incubation at 25 ºC/20 ºC (day/night) using a 16-h photoperiod and then transplanted to larger pots containing 5 L of soil mixture made of sand, silt and peat (2:1:1, by vol.) and 8 g of NPK controlled release fertilizer Nutricote® 15-9-10 (2MgO) + ME. The pots were maintained under open-air conditions and irrigated when required to avoid water stress. Evaluation for broomrape resistance was made by counting the number of emerged broomrape shoots for each sunflower plant at the end of sunflower flowering. Plants were classified as resistant if they showed no emerged broomrapes and susceptible if they showed emerged broomrape shoots.

#### Plant growth conditions and phenotyping for the field assay

For the field assay, sunflower seeds were germinated and sown in small pots (7 × 7 × 7 cm) as indicated previously, with the exception that the broomrape population used was SP (race F_GV_). As for the pot assays, the plants were kept in a growth chamber for 20–25 days using the same conditions. After this period, they were transplanted to an infested field plot at the Institute for Sustainable Agriculture (IAS-CSIC, Córdoba, Spain) in which only race-F_GV_ experiments have been conducted since 1999. A basal application of 500 kg ha^−1^ of 8–15-15 NPK fertilizer was made before transplanting. The plants were also irrigated when required to avoid water stress. Evaluation for broomrape resistance was made as explained for the pots assay.

#### Plant growth conditions and phenotyping for the multi-pot tray assay

For the multi-pot tray assay (Rodríguez-Ojeda et al. 2013), the homogeneously resistant F_2:3_ families were evaluated in small pots containing 40 cm^3^ of infested substrate in a multi-pot tray. The substrate consisted of a mixture of sand and peat (1:1 by vol) to which sunflower broomrape seeds of the G_TK_ race at an approximate concentration of 100 seeds (approx. 0.15 mg) per cm^3^ of soil were added. The mixture was carefully shaken in a plastic bag to obtain a homogeneously infested substrate. Sunflower seeds were germinated in moistened filter paper and planted in the pots. The plants were grown in a growth chamber at 25/20 ºC (day/night) with a 16-h photoperiod and photon flux density of 300 µmol m^− 2^ s^− 1^ for 60 days, after which they were uprooted and cleaned for counting the number of sunflower broomrape attachments on the roots (Rodríguez-Ojeda et al. 2013).

#### Plant phenotyping score

For the molecular study and *Or*_*Deb2*_ mapping, F_2_ plants were scored as follows. For race G_TK_, F_2_ plants were scored as homozygous dominant *Or*_*Deb2*_*Or*_*Deb2*_ if they were resistant and showed uniformly resistant plants in their respective F_3_ progeny (considering both the evaluations in 5L pots and in multi-pot tray assays), heterozygous *Or*_*Deb2*_*or*_*Deb2*_ if they were resistant and their F_3_ progeny segregated (i.e., showed both resistant and susceptible plants) and homozygous recessive *or*_*Deb2*_*or*_*Deb2*_ if they were susceptible and showed susceptible plants only in their respective F_3_ progeny. For race F_GV_, F_2_ plants were scored as described above for race G_TK_, considering the evaluations for this race F_GV_ in each F_2:3_ family. A chi-square test was used to evaluate the proposed one-gene segregation ratio for the population used in the molecular study.

#### Tissue sampling, gDNA extraction, and bulked segregant analysis

As described above, the mapping population consisted in an F_2_ population of 232 F_2_ plants phenotyped for race G_TK_, and for races F_GV_ and G_TK_ in their corresponding F_2:3_ families. Two fully expanded leaves from each F_2_ plant and the parental lines were cut and frozen at -80ºC. The leaf tissue was lyophilized and ground to a fine powder in a laboratory mill. gDNA was extracted as described in Pérez-Vich et al. (2004).

For bulked segregant analysis (BSA, Michelmore et al. 1991), bulks were constructed by pooling aliquots (30 μl) of gDNA from two sets of individuals with contrasting genotypes for the *Or*_*Deb2*_ gene based on phenotypic analyses for broomrape resistance of both F_2_ and F_3_ plant generations. The susceptible bulk was made up from 21 F_2_ individuals classified as *or*_*Deb2*_*or*_*Deb2*_ (homozygous susceptible) and the resistant bulk was made up from 21 individuals classified as *Or*_*Deb2*_*Or*_*Deb2*_ (homozygous resistant), as described above. A sunflower proprietary 600 k AXIOM® array containing 586,985 SNPs developed at LIPME-INRAE (Toulouse, France) in the frame of the SUNRISE project (ANR-11-BTBR-0005) was used to genotype the two bulks and the two parental lines IAS-31 and DEB2.

#### SSR genotyping

After confirming the location of the *Or*_*Deb2*_ gene on Chr4 through BSA, a set of SRR markers from this Chr4, mapped by Tang et al. (2002) and Yu et al. (2003) and identified by ORS and CRT prefixes, was screened for polymorphisms between the parental lines DEB2 and IAS-31. PCR analyses were performed as described by Pérez-Vich et al. (2004). SSR amplification products were separated on 3% (w/v) Metaphor® (BMA, Rockland, ME, USA) in 1 × TBE buffer with SaveView Nucleic Acid Stain (NBS Biologicals Ltd., Huntingdon, UK) incorporated in the gels and visualized under UV light. A 100 bp DNA ladder (Solis BioDyne, Tartu, Estonia) was used as a standard molecular weight marker to get an approximate size of DNA fragments. Bands were scored manually using Quantity One® 1-D Analysis Software (Bio-Rad Laboratories Inc, Hercules, CA, USA). SSR markers revealing polymorphisms, especially co-dominant, were then genotyped in the complete mapping population, following the protocols mentioned above.

#### SNP genotyping

Two sets of SNP markers from Chr4 were used, all selected based on the *Or*_*Deb2*_ position (upper half of Chr4) determined by BSA and within the first SSR genotyping assay. The first one consisted in SNP markers developed and mapped by Bachlava et al. (2012) and Bowers et al. (2012), identified by SFW prefixes. The second set was selected from the AXIOM markers identified as polymorphic between the resistant and the susceptible bulks. All these SNP markers were genotyped in a subset of the mapping population (ninety-two individuals) using competitive allele-specific PCR assays based on KASP™ technology (LGC genomics, Teddington, Middlesex, UK) at LGC genomics. In order to refine their position, the SNP markers mapping closest to *Or*_*Deb2*_ were then further genotyped in the complete mapping population (232 individuals).

#### Resistance gene candidate (RGC) genotyping

Since some RGC homologs to intracellular Nucleotide-binding Leucine-Rich Repeat (NLR or NB-LRR) proteins, which consist in a central NB domain and a C-terminal LRR, have been reported to map to the upper half of Chr4 (Radwan et al. 2008); they were used to develop more SNP markers. These NB-LRR RGC sequences were as follows: RGC41 (GenBank accession EF559382), RGC126 (EF559405), RGC127 (EF559406), RGC128 (EF559407), RGC129 (EF559408), RGC130 (EF559409), RGC133 (EF559412), RGC225 (EF559430), and RGC246 (EF559450). Primers were designed from these sequences with Lasergene SeqBuilder Pro software within the DNASTAR, Inc. package (Table S1) and used to amplify the parental lines IAS-31 and DEB2. PCR reactions were performed using 30 µl of reaction mixture containing 1 × PCR buffer, 1.5 mM MgCl2, 0.2 mM of dNTPs, 0.3 µM of 3′ and 5′-end primers, 0.7 units of Taq DNA polymerase (Biotaq™ DNA Polymerase, Bioline, London, UK), and 50 ng of genomic DNA. DNA amplification was performed in a GeneAmp PCR System 9700 (Applied Biosystems, Foster City, CA, USA) with an initial denaturation step at 91 °C for 5 min, followed by 40 cycles of 1 min at 91 °C, 1 min at the annealing temperature for each primer (48 to 58 °C), and 2 min at 72 °C, and ending with an extension period of 5 min at 72 °C. The amplification products were separated on a 1.5% agarose gel and purified by means of the QIAquick gel extraction kit (Qiagen GmbH, Hilden, Germany) for cloning, sequencing and SNP marker development. The purified fragments were ligated into the T/A vector pCR2.1 and the recombinants were transformed to TOP10 Chemically Competent *E. coli* using the TOPO-TA cloning kit (Invitrogen, San Diego, CA, USA) as described by the manufacturer. From five to ten recombinant bacterial colonies (white) per PCR product were picked from the plate containing ampicillin and X-gal as selective media and screened through PCR colony. Selected colonies were cultured overnight at 37 °C. Plasmids were extracted and purified using QIAprep Spin Miniprep Kit (Qiagen GmbH, Hilden, Germany). Restriction enzyme digestion was performed to further confirm the presence and size of the insert. Sequencing in both forward and reverse orientations of the cloned fragments (several clones per locus, as described in the results section) was carried out using the M13 forward and reverse sequencing primers (Stab Vida, Lisbon, Portugal). Sequences were verified to be from genes encoding sunflower NB-LRR type proteins through BLAST analyses. Sequence alignments for each RGC were carried out and used to group the sequences and to identify clusters, corresponding to different loci, since NB-LRR encoding genes are frequently duplicated. Sequences of the resistant (DEB2) and the susceptible (IAS-31) parental lines from the same locus were analyzed for DNA polymorphisms, and gene-specific molecular markers for the SNP polymorphisms found were genotyped using KASP™ assays, as described above. Sequence analysis was conducted with the aid of the Lasergene SeqMan Ultra and MegAlign Pro software within the DNASTAR, Inc. package.

### Genetic linkage analysis, Or_Deb2_ mapping, and candidate gene analysis

Genetic linkage analysis was run with MAPMAKER v.3.0 (Whitehead Institute, Cambridge, MA; Lander et al. 1987) using segregation data for SSRs, SNPs, and RGC-SNPs, as well as for *Or*_*Deb2*_ gene. The genotypes for *Or*_*Deb2*_ gene were inferred from their corresponding phenotypes based on the F_2_ and F_2:3_ evaluations for broomrape resistance to races G_TK_ and F_GV_, as described above. Two-point analysis was used to group the marker loci. A LOD threshold of 6 and a maximum recombination fraction of 0.3 were used as linkage criteria. Three-point and multi-point analyses were used to determine the order and interval distances between the markers. Recombination fractions were converted to centiMorgans (cM) using the Kosambi mapping function. Linkage group maps were drawn using the MapChart software (Voorrips 2002). Linkage analyses were performed in consecutive SSR, RGC-SNP, and SNP marker genotyping rounds, each one reducing the *Or*_*Deb2*_ mapping interval, as explained in the results section. A final linkage analysis was carried out using genotyping data from the complete mapping population of the closest markers to the *Or*_*Deb2*_ gene, in order to refine their position.

For the candidate gene analysis, *Or*_*Deb2*_ closest markers were mapped on the HanXRQr2.0-SUNRISE reference sunflower genome sequence (https://www.heliagene.org/HanXRQr2.0-SUNRISE). Positions in the previous sunflower genome assembly (https://www.heliagene.org/HanXRQ-SUNRISE) were also determined in order to compare with previous reports in which only this assembly was used. After the physical positions were extracted, the genomic region delimited by the *Or*_*Deb2*_ flanking markers was examined to identify the annotated protein-coding genes. The nature of the most significant annotated candidate genes, and of all the genes coding for uncharacterized proteins, genes with unknown function or genes directly annotated but without description was also verified in the NCBI *Helianthus annuus* annotation release 101 (2020-09-02), and through BLAST searches using the sunflower nucleotide and amino acid sequences. The genomic region delimited by the *Or*_*Deb2*_ flanking markers was also explored for the existence of gene clusters of tandem duplicated genes, as this has been reported for the genomic organizations of many major resistance genes. Tandem duplicated genes were defined as those closely related in the same gene family and clustered together (Cannon et al. 2004), and established using the following parameters: a cluster must contain at least two genes, the distance between two neighboring genes should be < 200 kb, and no more than eight genes should be present between the neighboring genes (Jupe et al. 2012). Finally, for specific protein coding genes, detailed in the results section, protein domains were analyzed by the InterProScan program (http://www.ebi.ac.uk/Tools/pfa/iprscan/), and homology between amino acid sequences was calculated using Lasergene MegAlign Pro software within the DNASTAR, Inc. package. Multiple sequence alignments were generated by using the Clustal omega program, and a phylogenetic tree was constructed by maximum likelihood (RAxML) algorithm.

### Characterization of resistance mechanisms

To ascertain whether the DEB2 resistance was based in pre-attachment or post-attachment mechanisms, two types of experiments were conducted. The in vitro germination experiments were used to identify allelopathic action against *O. cumana* germination and the rhizotron experiments were used to identify post-attachment mechanisms during the *O. cumana* infection process.

#### In vitro experiments for analysis of germination induction activity

The root activity of sunflower line DEB2 on broomrape seed germination was compared with the root activity of sunflower lines B117, NR5 and P96 using an in vitro germination experiment (Fernández-Aparicio et al. 2014). Seeds of two broomrape species: *Orobanche cumana*, population collected in Guadalquivir Valley (southern Spain), and *Phelipanche ramosa*, population collected in southern France, were used to identify differences in germination induction activity for each sunflower line.

Sunflower seeds were surface sterilized with 4% sodium hypochlorite containing 0.02% (v:v) Tween 20, rinsed three times with sterile distilled water and placed on moistened filter paper inside Petri dishes to allow germination. After four days, germinated sunflower seeds were transferred to pots filled with sterile perlite in a growth chamber (23/20 °C, 16/8 h day/night). Plants received Hoagland’s nutrient solution (Hoagland and Arnon 1950) modified at one-quarter strength twice per week. Sunflower plants were removed from the perlite, their roots carefully washed and individually placed in tubes by immersing the roots for 24 h in sterile distilled water, allowing them to release the root exudates. The solutions containing the sunflower root exudate were collected, and the total sunflower root contained in each tube weighed. In order to make valid comparisons across sunflower lines and plants, root exudate solution was adjusted with sterile distilled water to achieve equivalent concentrations of 0.02 and 0.01 g of sunflower root fresh weight /mL of hydroponic media (root exudate solution).

Broomrape seeds were surface sterilized by immersion in 0.5% sodium hypochlorite containing 0.02% (v:v) Tween 20, for 5 min, rinsed thoroughly with sterile distilled water, and dried in a laminar air flow cabinet. Approximately 100 seeds of each broomrape species were placed separately in 9 mm diameter glass fiber filter paper discs (GFFP) (Whatman International Ltd., Maidstone, UK) moistened with 50 μL of sterile distilled water and placed inside Petri dishes in incubators at 23 ºC during 10 days to allow seed conditioning. GFFP discs containing conditioned seeds of each broomrape species were transferred onto a sterile sheet of paper to remove the excess of water and transferred to new 10 cm sterile Petri dishes. Differences in germination induction were studied by applying triplicate aliquots of 50 μL of root exudate collected from each sunflower plant (four plants per sunflower line) at each harvesting time. Sterile distilled water was used as negative control. GR24 (10^−6^ M) was used as a positive control. Seeds were stored in the dark at 23 °C for 7 d to allow germination. The germination was scored for each GFFP disc by determining the number of germinated seeds on 100 seeds using a stereoscopic microscope. Seeds were considered germinated when radicle was visible through the seed coat.

#### Rhizotron experiment for analysis of post-attachment resistance responses

The interaction of roots of sunflower line DEB2 with seedlings of the three *O. cumana* populations: OC-94 (race E_GV_), SP (race F_GV_), and GT (race G_TK_) was studied using rhizotron experiments (Fernández-Aparicio et al. 2008). The DEB2 responses to each *O. cumana* population were compared to the responses of sunflower lines B117, NR5, and P96.

Sunflower seeds were surface sterilized with 2% sodium hypochlorite containing 0.02% (v:v) Tween 20 for 5 min and then rinsed thoroughly with sterile distilled water and germinated in 9 cm diameter petri dishes with moistened filter papers placed for 4 days in a growth chamber under dark warm (23 °C) conditions before the setting of each experiment. Nine sunflower seedlings per sunflower line were individually transferred to GFFP sheets and placed over square Petri dishes (12 cm by 12 cm) filled with sterile perlite moistened with sterile distilled water. Petri dishes were previously punctured on the top to allow sunflower stem develop outside of the dish. Seeds of OC-94, SP and GT populations of *O. cumana* were surface sterilized by immersion in 0.5% sodium hypochlorite containing 0.02% (v:v) Tween 20, for 5 min, rinsed thoroughly with sterile distilled water, spread separately over GFFP sheets (12 sheets for each population) at a density of 50 seeds cm^2^ and stored in the dark for 10 days to allow *O. cumana* seed conditioning. Then, the GFFP sheets containing the roots of each sunflower line were replaced by GFFP sheets containing the conditioned *O. cumana* seeds (3 GFFP sheets of each *O. cumana* population per sunflower line) allowing simultaneous reception of *O. cumana* germination stimulants in the seeds. The Petri dishes containing sunflower-*O. cumana* co-cultivation system were sealed with parafilm, wrapped in aluminum foil and stored vertically in a growth chamber (23/20 °C, 16/8 h day/night). Plants received Hoagland’s nutrient solution (Hoagland and Arnon 1950) modified at one-quarter strength twice per week. *O. cumana* seeds located at a distance of 3 mm from the sunflower roots were inspected under a stereoscopic microscope to determine (i) the percent of contacted *O. cumana* radicles that successfully penetrated sunflower root and formed a healthy tubercle and (ii) total number of *O. cumana* tubercles per sunflower plant.

An additional rhizotron experiment was conducted to confirm the resistance response of DEB2 against the most virulent *O. cumana* population GT. The response of DEB2 against the infection of *O. cumana* population GT was compared with that of the B117 susceptible line. Six plants of each sunflower line were co-cultivated in rhizotron with *O. cumana* population GT as described above. *O. cumana* seeds located at a distance of 3 mm from the sunflower roots were inspected under a stereoscopic microscope to determine (i) percentage of *O. cumana* germination (ii) percentage of *O. cumana* germinated seeds whose radicles oriented towards the sunflower root and made contact with sunflower root surface (iii) percentage of radicles attached to sunflower root surface which successful penetrated and formed a healthy tubercle as consequence of successful nutrient transfer (iv) total number of *O. cumana* tubercles per sunflower plant.

#### Histopathological study

A dedicated rhizotron study was performed to obtain samples for the histological study of the interaction of DEB2 and B117 with the most virulent *O. cumana* population GT. Six plants of each sunflower line were co-cultivated in rhizotron with *O. cumana* population GT as described above. For each sunflower plant, ten randomly chosen sunflower root pieces of 0.5 cm length carrying attached *O. cumana* seedlings were cut under stereoscopic microscope and placed in FAE solution (formalin, acetic acid, 95% ethanol, and distilled water [10:5:50:35 vol/vol/vol/vol]) for at least 48 h. Then, the sunflower- *O. cumana* samples were dehydrated in a tertiary butyl alcohol series (70, 85, 90, 100%) and embedded in paraffin (58 °C melting point; Merck, Darmstadt, Germany). Paraffin-embedded tissues were sectioned in orientation transversal to the sunflower vascular system and longitudinal to vascular system of the parasite. The sections were obtained with a rotary microtome at 8 μm and immediately attached to adhesive-treated glass slides. After removal of paraffin, the sections were stained with a combination of tannic acid–ferric chloride, safranin, and fast green, by which nuclei, chromosomes, and lignified or suberized cell walls stain red, cytoplasm and cellulosic cell walls stain green, and the tannic acid–iron chloride aids in cell wall definition and is considered to be a general test for phenols (Jensen 1962; Reeve 1951; Ruzin 1999; Palomares-Rius et al. 2019). The stained tissue was evaluated under a light microscope to observe the process of parasitic invasion into sunflower tissues.

#### Statistical analysis

Experiments were performed using a completely randomized design. Percentage data were approximated to normal frequency distribution by means of angular transformation (180/Π × arcsine (sqrt[%/100]) and subjected to analysis of variance (ANOVA) using SPSS software. The significance of mean differences among treatments was evaluated by Tukey HSD test. Null hypothesis was rejected at the level of 0.05.

## Results

### Genetic mapping of the Or_Deb2_ gene

#### Phenotyping of the mapping population and bulked segregant analysis

The evaluation of the 278 F_2_ plants for race G_TK_ resistance showed 197 resistant plants and 81 susceptible plants. The observed ratio was not significantly different from the expected 3:1 (resistant: susceptible) ratio for the segregation of a dominant resistance gene (χ^2^ = 2.54, *P* = 0.11). In the evaluation for race G_TK_ of the 232 progenies from the mapping population, 49 F_2:3_ families showed resistance in all F_3_ plants, 75 families susceptibility in all F_3_ plants, and 108 families segregation for resistance (i.e., with both resistant and susceptible F_3_ plants). From these 232, a total of 220 F_2:3_ families were also evaluated for race F_GV_, which resulted in 49 families consistently resistant, 99 segregating, and 72 consistently susceptible. Comparison between the evaluation for both F_GV_ and G_TK_ races in these 220 F_3_ families (screened against both races) showed identical results, with a complete phenotypic correspondence and family classification (as homogeneously susceptible, segregating or homogeneously resistant). The classification of the F_2_ genotypes from the mapping population (n = 232) based on the evaluation of their F_3_ families was slightly different from a 1:2:1 ratio (resistant: segregating: susceptible) in the evaluations conducted with race G_TK_ (n = 232; ratio 49:108:75; χ^2^ = 6.93, *P* = 0.03) and F_GV_ (n = 220, ratio 49: 99: 72; χ^2^ = 7.00, *P* = 0.03). It was observed an excess of homozygous susceptible progenies, mainly at the expense of the number of homozygous resistant progenies.

For bulked segregant analysis, the parental lines (DEB2 and IAS-31), and the resistant and susceptible bulks were genotyped with the AXIOM array. From the 586,985 SNPs, 67,374 were homozygous and polymorphic between DEB2 and IAS-31, of which 563 were also homozygous polymorphic between the bulks and showed the same genotypes between the resistant bulk and the DEB2 line and between the susceptible bulk and the IAS-31 line. From these 563 SNPs, 543 were aligned perfectly and uniquely on the HanXRQr2.0 genome sequence and 519 of them were located on Chr4, 36% (189 SNPs) being mapped in the 4% of the Chr4’s size (the first 10 Mb).

### Or_Deb2_ mapping

**Fig. 1:**
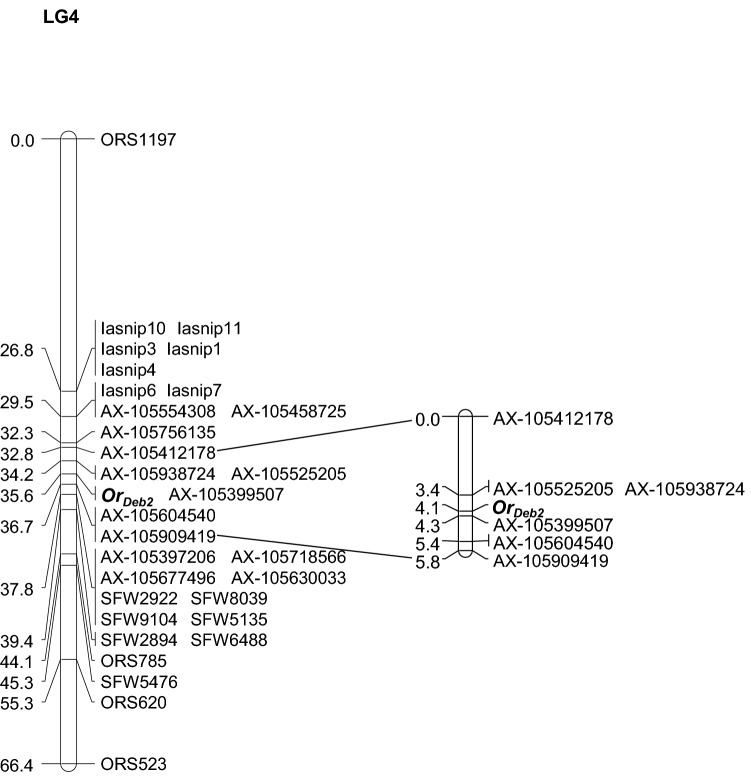
Linkage map of sunflower chromosome (Chr) 4 containing the *Or*_*Deb*2_ gene. The ORS prefix denote SSR marker loci; the SFW prefix, SNP marker loci mapped by Bowers et al. (2012); the AX prefix, SNP marker loci from the 600 k AXIOM® array developed at LIPME-INRAE (Toulouse, France); and the Iasnip prefix SNP markers developed in this study from sunflower NB-LRR resistance gene candidates. Left figure: complete map; right figure: refined map of the *Or*_*Deb*2_ region. The cumulative distances in centiMorgans are shown at the left of each map

A first genotyping was performed with the Chr4 SSR selected from public maps (Tang et al. 2002, 2003) and RGC-SNPs developed in this research as follows. From a total of 26 primer pairs (Table S1) designed from Chr4 RGC homologs (Radwan et al. 2008), clear amplification products which when sequenced could be grouped into a single contig (corresponding to one locus) were obtained for RGC127, RGC130 and RGC246. Blast alignments on the sunflower genome (HanXRQr2.0-SUNRISE) revealed high homology (> 95%) with Chr4 genes coding for the disease resistance protein RUN1 and TMV resistance protein N, both of them being members of the Toll-interleukin-1 receptor/nucleotide-binding site/leucine-rich repeat (TIR-NBS-LRR) class of plant resistance genes (Table S2), excepting for RGC246, which showed no significant hits and was excluded for mapping. SNP polymorphisms between the parental lines were identified for each locus and allowed design of the SNP markers Iasnip-1, 3, 4, 10 and 11 for RGC-127, and Iasnip-6 and 7 for RGC-130 (Table S2), which were genotyped in the complete mapping population. This enabled to map *Or*_*Deb2*_ between a group of RGC-SNP markers [Iasnip-1, 3, 4, 10 and 11 (from RGC-127), and Iasnip-6 and 7 (from RGC-130)] and the SSR marker ORS785, at genetic distances of 8.1 (RGC-127-SNPs) and 5.3 cM (RGC-130-SNPs) in the *Or*_*Deb2*_ distal position, and of 11.7 cM in the *Or*_*Deb2*_ proximal region (ORS785). A second round of genotyping was made in 92 individuals with 18 SFW SNP markers found in this interval (Bowers et al. 2012), which narrowed the *Or*_*Deb2*_ interval between RGC-130-SNP markers (Iasnip6 and 7; physical position 4.05 Mbp, Table S4) and a group of three SFW markers (SFW2922, SFW8039, and SFW9104; physical position from 16.2 to 18.0 Mbp, Table S4), which were located 5.3 cM distal and 2.2 cM proximal, respectively, to *Or*_*Deb2*_. A total of 18 AXIOM markers (Table S3) polymorphic between the bulks and located within this genomic interval were further genotyped in 92 individuals, which allowed the final construction of the Chr4 linkage map containing the *Or*_*Deb2*_ gene (Fig. 1, left). *Or*_*Deb2*_ location was refined by genotyping the complete mapping population (232 individuals) using the six SNP makers closest to *Or*_*Deb2*_, which determined mapping position of *Or*_*Deb2*_ between AXIOM SNP markers AX-105525205/AX-105938724 and AX-105399507, at 0.7 and 0.2 cM, respectively (Fig. 1, right).

**Fig. 2:**
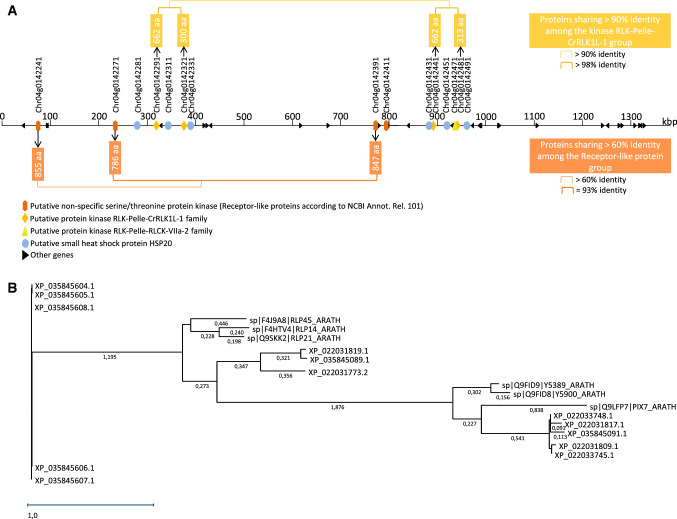
**a** Gene organization in the *Or*_*Deb2*_−1.38 Mbp region delimited by SNP markers AX-105525205 and AX-105399507 flanking *Or*_*Deb2*_ (physical positions from 7,892,288 bp to 9,272,600 bp of the XRQr2.0 assembly). The most abundant gene products in this interval are highlighted in colors, and the others are indicated in black. Details of all the genes in this interval are found in Table 2 and Table S5. Distances are indicated as kbp. Similarity between closely related genes based on pairwise amino acid sequence comparisons is also indicated; **b** Phylogenetic relationships among protein kinase genes in the *Or*_*Deb2*_-1.38 Mbp interval inferred by maximum likelihood from amino acid sequence alignment using the MegAlign Pro V17 sequence analysis software. Related Arabidopsis thaliana genes are also included. Sunflower protein sequences used for the analysis and their corresponding loci in the XRQr2.0 assembly, and the NCBI Helianthus annuus annotation release 101 annotations are detailed in Table S5. Arabidopsis protein sequences and their corresponding loci are as follows: Q9FID9/Y5389_ARATH: Probable receptor-like protein kinase At5g38990; Q9FID8/Y5900_ARATH Putative receptor-like protein kinase At5g39000; Q9LFP7/PIX7_ARATH Probable serine/threonine-protein kinase PIX7; F4HTV4/RLP14_ARATH Receptor-like protein 14 At1g74180; Q9SKK2/RLP21_ARATH Receptor-like protein 21 At2g25470; F4J9A8/RLP45_ARATH Receptor-like protein 45 At3g53240

The markers flanking *Or*_*Deb2*_ (AX-105525205/AX-105938724 and AX-105399507) delineated a window of 4.6 Mbp between physical positions 10,500,940 bp and 15,126,594 bp of the XRQr1.0 sunflower genome assembly (https://www.heliagene.org/HanXRQ-SUNRISE; Badouin et al. 2017) (Table S4). This window differed in the latest and improved XRQr2.0 sunflower genome assembly (https://www.heliagene.org/HanXRQr2.0-SUNRISE) (Table S4). In this assembly, AX-105938724 and AX-105525205 mapped at physical positions 7,880,416 bp and 7,892,288 bp, respectively, and blast searches for the AX-105399507 context sequence revealed no hits (Table S4). However, XRQr2.0 blast searches of the XRQr1.0 sequences flanking the AX-105399507 context sequence in a 76 kb window revealed that AX-105399507 was inside a gap in the XRQr2.0 assembly found between positions 9,272,600 and 9,317,035 bp. This delineated a window for *Or*_*Deb2*_ of 1.38 Mbp between physical positions 7,892,288 bp and 9,272,600 bp of the XRQr2.0 assembly. Exploration of this *Or*_*Deb2*_-1.38 Mbp genomic region revealed a total of 41 annotated genes (Table 2 and Fig. 2a). Among them, the most abundant gene products were protein kinases (9 out of 41, 22%), followed by small heat shock proteins HSP20 (6 out of 41, 14.6%) (Table 2 and Fig. 2a). Protein kinase genes belonged to two groups (Fig. 2b), as it will be discussed below. These were annotated as (i) putative non-specific serine/threonine protein kinases (four genes) [receptor-like proteins (RLPs) according to NCBI annotation release 101], and (ii) protein kinases of the RLK (Receptor-Like Kinase)-Pelle class (five genes) (receptor-like proteins kinases or serine/threonine protein kinases according to NCBI annotation release 101) (Table S5 and Fig. 2a).

**Table 2:** Genes found within the HanXRQChr04 (HanXRQr2.0 assembly) *Or*_*Deb2*_−1.38 Mbp region delimited by SNP markers AX-105525205 and AX-105399507

Position HanXRQr2 Start	Position HanXRQr2 End	HanXRQr2 Gene identification	HanXRQr2 description
7,937,879	7,939,128	Chr04g0142231	Hypothetical protein
7,967,895	7,970,173	Chr04g0142241	**Putative non-specific serine/threonine protein kinase**
7,985,963	7,986,763	Chr04g0142251	Putative polygalacturonase
7,990,551	7,992,626	Chr04g0142261	
8,125,286	8,127,830	Chr04g0142271	**Putative non-specific serine/threonine protein kinase**
8,173,923	8,175,953	Chr04g0142281	Putative small heat shock protein HSP20
8,209,577	8,213,256	Chr04g0142291	**Putative protein kinase RLK-Pelle-CrRLK1L-1 family**
8,220,399	8,221,408	Chr04g0142301	Hypothetical protein
8,239,405	8,239,779	Chr04g0142311	Putative small heat shock protein HSP20
8,266,400	8,267,304	Chr04g0142321	**Putative protein kinase RLK-Pelle-CrRLK1L-1 family**
8,285,420	8,285,776	Chr04g0142331	Putative small heat shock protein HSP20
8,313,884	8,314,515	Chr04g0142341	Putative acyl-CoA desaturase
8,320,071	8,320,651	Chr04g0142351	Putative acyl-CoA desaturase
8,322,397	8,324,950	Chr04g0142361	Putative protein
8,512,036	8,513,088	Chr04g0142371	Hypothetical protein
8,566,829	8,569,474	Chr04g0142381	Putative cation/H + exchange, CPA1 family, na + /H + exchanger NHX -type
8,664,186	8,666,345	Chr04g0142391	**Putative non-specific serine/threonine protein kinase**
8,673,302	8,675,025	Chr04g0142401	Hypothetical protein
8,687,041	8,712,011	Chr04g0142411	**Putative non-specific serine/threonine protein kinase**
8,728,796	8,729,206	Chr04g0142421	Putative ribosomal protein S2
8,774,521	8,774,877	Chr04g0142431	Putative small heat shock protein HSP20
8,779,670	8,783,100	Chr04g0142441	**Putative protein kinase RLK-Pelle-CrRLK1L-1 family**
8,817,610	8,817,966	Chr04g0142451	Putative small heat shock protein HSP20
8,818,793	8,818,868	Chr04g0142461	tRNA-Val
8,820,857	8,821,668	Chr04g0142471	**Putative protein kinase RLK-Pelle-RLCK-VIIa-2 family**
8,826,964	8,827,974	Chr04g0142481	**Putative protein kinase RLK-Pelle-CrRLK1L-1 family**
8,856,647	8,857,083	Chr04g0142491	Putative small heat shock protein HSP20
8,859,823	8,860,883	Chr04g0142501	Hypothetical protein
8,874,922	8,875,499	Chr04g0142511	Hypothetical protein
8,877,476	8,878,997	Chr04g0142521	Hypothetical protein
8,918,927	8,920,006	Chr04g0142531	Putative RNA-directed DNA polymerase
8,920,828	8,921,630	Chr04g0142541	Putative RNA-directed DNA polymerase
8,996,833	8,999,227	Chr04g0142551	Putative acyl-CoA desaturase
9,130,932	9,133,371	Chr04g0142561	Putative acyl-CoA desaturase
9,138,335	9,138,731	Chr04g0142571	Putative ubiquitin-conjugating enzyme E2, ubiquitin-conjugating enzyme/RWD
9,163,287	9,167,722	Chr04g0142581	Putative ubiquitin-conjugating enzyme E2, ubiquitin-conjugating enzyme/RWD
9,169,246	9,173,597	Chr04g0142591	Hypothetical protein
9,198,801	9,200,039	Chr04g0142601	Putative RNA-directed DNA polymerase
9,206,248	9,219,653	Chr04g0142611	Putative ribonuclease H-like superfamily, PRO8NT domain, PROCN domain, PROCT domain, MPN
9,209,240	9,210,095	Chr04g0142621	Hypothetical protein
9,219,071	9,219,563	Chr04g0142631	Hypothetical protein

Putative candidate genes for *Or*_*Deb2*_ are highlighted

Gene organization within the *Or*_*Deb2*_-1.38 Mbp region was explored, and two putative subregions (from positions 200 to 400 and from 750 to 1000 kbp) were observed (Fig. 2a) according to gene order and amino acid sequence identity of the gene products (Fig. 2a, b). Each group contained at least one gene classified as RLP- “putative non-specific serine/threonine protein kinases,” two highly similar (> 98% amino acid identity) tandemly duplicated kinase genes classified within the RLK-Pelle class, and three small heat shock proteins. Amino acid sequence identity of the gene products belonging to the RLP or the RLK-Pelle class between these two regions was > 90% (excepting for Chr04g0142411) (Fig. 2).

Since the only gene conferring resistance to *O. cumana* cloned to date is a receptor-like kinase (Duriez et al. 2019), the nature of the receptor-like proteins and the protein kinase genes in the *Or*_*Deb2*_-1.38 Mbp region, their domain constitution and similarity with corresponding genes were further examined. Four of the five protein kinases of the RLK-Pelle class belonged to the *Cr*RLK1L-1 (*Catharantus roseus* RLK-like kinase-like) family (Fig. 2a). TAIR BlastP analyses of these four kinases showed the Arabidopsis (*Arabidopsis thaliana*) homologues At5g39000 and At5g38900 as those with the best hits (e-values lower than 1 × 10^−70^) (Fig. S1), both being members of the *Cr*RLK1L-1 kinase class. These two Arabidopsis genes are tightly linked on the Arabidopsis genome and have been shown to be regulated upon bacterial infection or bacterial and oomycete elicitor treatments (Lindner et al. 2012) and to be involved in mediating adaptation to metal ions stress (Richter et al. 2018). It was observed that within the two sunflower *Cr*RLK1L-1 kinase pairs sharing > 98 amino acid identity (Chr04g0142291/Chr04g0142321; and Chr04g0142441/Chr04g0142481), sequence length was doubled in one protein with respect to other (662aa/300aa and 662aa/313aa, respectively) (Fig. 2a; Fig S1). This duplication was also observed at the protein domain and homology level, since there were identified two kinase domains (Prosite PS50011), each spanning around a half of the protein, in those with about 600 aa, and only one in those with around 300 aa (Fig. S1). TAIR BlastP analyses showed that each half of the longer proteins had homology to the kinase region of At5g38900 or At5g38900 (Fig. S1). However, a unique serine/threonine active site (InterPro IPR008271) (Ser/Thr AS) and ATP binding site (ATP-BS) (InterPro IPR017441) were present in the four proteins (Fig. S1). In the longer (about 600 aa) *Cr*RLK1L-1 kinases, these unique Ser/Thr AS and ATP-BS were located in the left-kinase domain which showed higher homology with the kinase region of the Arabidopsis genes than the right domain (Fig. S1). No transmembrane or extracellular domains were observed in any of the four RLK-Pelle-CrRLK1L-1 kinases (Fig. S1). PANTHER family classification system grouped the four sunflower *Cr*RLK1L-1 kinase genes in the OS07G0166700 protein family (PTHR27003) [Os07g0166700 (LOC434248) encodes a putative brassinosteroid LRR receptor kinase] (Fig. S1). Within a total of 37 plant species with genes assigned to this family, sunflower was by far the one with the highest number of genes represented (420, compared to 20 in Arabidopsis), followed by other Compositae species, lettuce (*Lactuca sativa*) with 245, being the rest with about or less than 100 genes represented. Within the PTHR27003 family, the four sunflower *Cr*RLK1L-1 kinase genes in the *Or*_*Deb2*_-1.38 Mbp interval were additionally assigned to subfamily PTHR27003:SF342 (serine-threonine/tyrosine-protein kinase catalytic domain-containing protein-related, which also integrated subfamily SF345) which contained only genes from sunflower (26 genes, all having one or two kinase domains and lacking extracellular domains) and lettuce (20 genes). Interestingly, the majority of the sunflower kinase genes in SF342 (17 out of 26) were located in Chr 4, very close to the *Or*_*Deb2*_*-*1.38 Mbp region (12 of them were located in a window from 6.5 to 7.4 Mbp, 4 were the CrRLK1L-1 kinase genes in the *Or*_*Deb*2_-1.38 Mbp region, located from 8.2 to 8.7 Mbp, and one of them was located at 11.5 Mbp). BlastP searches against the non-redundant (nr) GenBank protein database revealed the best matching putative homologues to the four sunflower RLK-Pelle-CrRLK1L-1 kinases in other plant species to be two *Lactuca sativa* receptor-like protein kinase FERONIA proteins (LOC111885056 and LOC111896681) (e-values lower than 1 × 10^−100^), which had also two kinase domains (Prosite PS50011), one serine/threonine active site (InterPro IPR008271) and one ATP binding site (InterPro IPR017441), and lacked any transmembrane or extracellular domains.

The four protein coding genes in the other group were classified as putative non-specific serine/threonine protein kinases [receptor-like proteins (RLPs) according to NCBI Annot. Rel. 101] (Fig. 2a, b). TAIR BlastP analyses of these proteins showed them to be homologous to Arabidopsis RLP-15 (At1g74190) (for Chr04g0142241), RLP-14 (At1g74180) and RLP-21 (At2g25470) (for Chr04g0142271), and RLP-21 (for Chr04g0142391 and Chr04g0142411) (e-values lower than 1 × 10^−130^, excepting for Chr04g0142241 with e-values around 1 × 10^−26^) (Fig. S1). RLPs have been shown to be implicated in plant growth and development as well as in pathogen defense (Wang et al. 2010). Although the biological functions of most AtRLP genes still remain unknown, a number of AtRLP genes have assigned functions in disease resistance (AtRLP3/RFO, Shen and Diener 2013; AtRPP27, Tör et al. 2004; AtRLP30 Wang et al. 2008; AtRLP51/SNC2 and AtRLP55, Zhang et al. 2010). Also, transcriptional regulation of the AtRLP21 gene upon exposure to the bacterial pathogen *Pseudomonas syringae* and bacterial patterns HrpZ and Flg22 has been described (Wu et al. 2016). InterPro analysis of the four sunflower RLPs revealed that they had a large LRR region (InterPro IPR032675, IPR001611) spanning the majority of the protein sequence which was predicted to be extracellular, and lacked an intracellular kinase domain (Fig. S1). Chr04g0142271 (Fig. S1) and Chr04g0142391, which were 93% identical, also showed small transmembrane and cytoplasmic domains at the C-terminus protein region, being the domain constitution very close to that of their homologous Arabidopsis RLP genes. PANTHER grouped these two genes and Chr04g0142241 in the “receptor-like protein 14” (PTHR48062) family, which was represented in sunflower by 17 genes. Again, the majority of these sunflower genes (14 out of 17) were located at Chr4, very close to the *Or*_*Deb2*_-1.38 Mbp region [1 of them was located at 7.85 Mbp, 3 were the RLP genes in the *Or*_*Deb2*_-1.38 Mbp region (located from 7.97 to 8.66 Mbp), and 10 of them were in a window from 9.35 to 10.78 Mbp]. BlastP searches against the non-redundant (nr) GenBank protein database revealed the best matching putative homologues to the three RLPs Chr04g0142241, Chr04g0142271 and Chr04g0142391 in other plant species to be the *Lactuca sativa* RLP 12 (LOC11190055) (e-value 0.0), which also had a large non-cytoplasmic LRR region and small transmembrane and cytoplasmic domains at the C-terminus protein region.

### Characterization of resistance mechanisms

#### In vitro experiment for the characterization of pre-attachment resistance responses

**Fig. 3:**
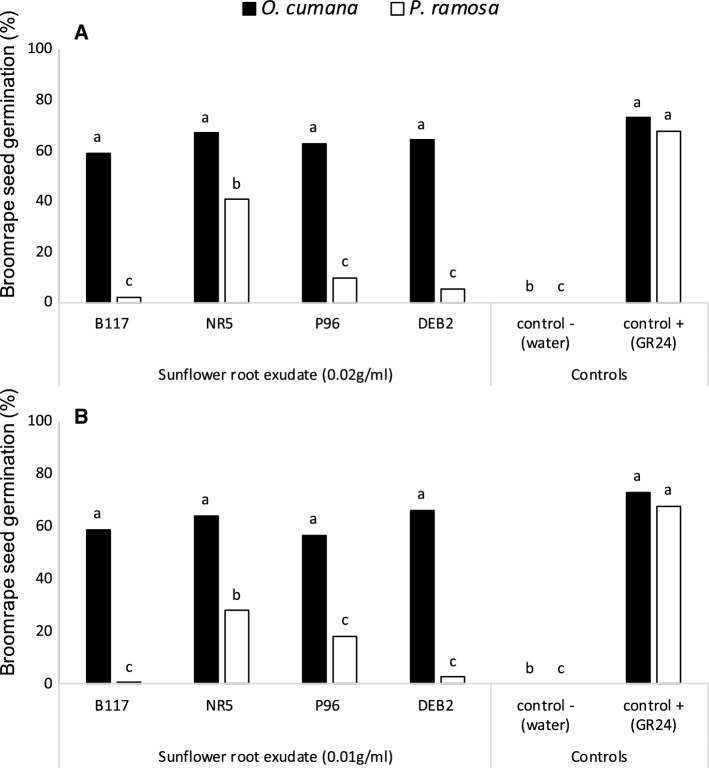
Effect of hydroponically collected root exudate from sunflower susceptible and resistant lines on stimulation of seed germination of seeds of *O. cumana* and *P. ramosa* at concentrations of **a** 0.02 g of sunflower root fresh weight /mL of root exudate solution; and **b** 0.01 g of sunflower root fresh weight /mL of root exudate solution. Analysis of variance was applied to transformed replicate data. For each treatment, bars with different letters are significantly different according to the Tukey test (*p* < 0.05)

The germination induction effect of DEB2 root exudates in comparison with those of sunflower lines B117, NR5, and P96 on seeds of *O. cumana* and *P. ramosa* is shown in Fig. 3. In all cases, null germination was observed when seeds of both broomrape species were treated with negative control (distilled water). Seed germination of the two species was induced by the synthetic strigolactone GR24 used as a positive control. Significant effects in broomrape seed germination were observed individually for broomrape species (*O. cumana* and *P. ramosa*) (ANOVA, *p* < 0.001), sunflower line (DEB2, B117, NR5, and P96) (ANOVA *p* < 0.001), root exudate concentration (0.02 and 0.01 g of sunflower root/ml of root solution), (ANOVA *p* < 0.001) and also for the interaction of sunflower line and broomrape species (ANOVA *p* = 0.004). The interaction of sunflower line with root exudate concentration, the interaction of broomrape species with root exudate concentration, and the triple interaction of sunflower line, broomrape species and root exudate concentration were not significant.

*O. cumana* germination was induced by the root exudates of all sunflower cultivars, at all concentrations tested (Fig. 3). Root exudates of DEB2 induced similar levels of *O. cumana* germination than the susceptible line B117. On the contrary, induction of *P. ramosa* germination was much lower than that of *O. cumana* and significantly different across sunflower lines (Fig. 3). While negligible levels of *P. ramosa* germination were induced by root exudates of the resistant sunflower line DEB2 but also by those of B117 and P96, at all concentrations tested, root exudates of sunflower line NR5 induced significantly higher levels of *P. ramosa* germination.

#### Rhizotron experiment for the characterization of post-attachment resistance responses

**Fig. 4:**
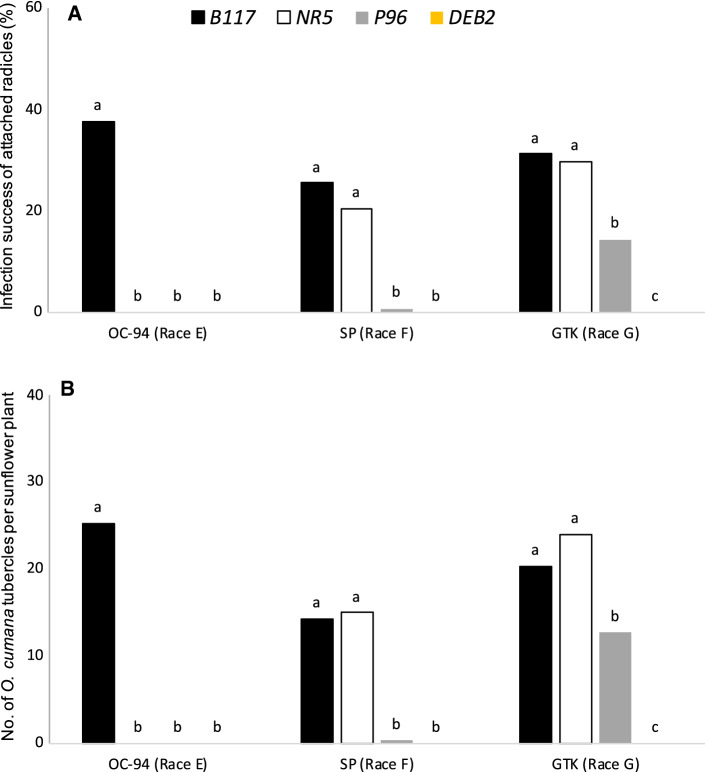
Differences between sunflower resistant line DEB2 and control lines B117 (susceptible to all *O. cumana* races), NR5 (susceptible to *O. cumana* race F_GV_) and P96 (susceptible to *O. cumana* race G_TK_) during the infection process of OC-94, SP, and GT (races E_GV_, F_GV_ and G_TK_ respectively). **a** Infection success of *O. cumana* attached radicles measured as the percent of attached radicles that formed tubercle; **b** number of *O. cumana* tubercles formed per sunflower plant

In a first rhizotron experiment, the response of DEB2 to infection of the three *O. cumana* populations, OC-94, SP, and GT (races E_GV_, F_GV_ and G_TK_ respectively), was compared to the response of three sunflower control lines B117 (susceptible to all *O. cumana* races), NR5 (susceptible to *O. cumana* race F_GV_) and P96 (susceptible to *O. cumana* races G from Eastern Europe countries, including G_TK_). To assess differences among *O. cumana* populations in infection capability, two characteristics were examined for each sunflower line: (i) percentage of sunflower-attached *O. cumana* radicles that formed tubercle and (ii) total number of *O. cumana* tubercles per sunflower plant. Significant effects were observed for *O. cumana* population and sunflower line in infection success of the attached radicles (ANOVA *p* < 0.001, ANOVA *p* < 0.001 respectively) and in total number of tubercles per plant (ANOVA *p* < 0.001, ANOVA *p* < 0.001, respectively). The infection success of all *O. cumana* populations was high in roots of B117, whereas was completely inhibited in roots of DEB2. This was related with the number of tubercles per plant. High numbers of tubercles were formed by the three *O. cumana* populations on B117 sunflower plants whereas infection of the three *O. cumana* populations was completely resisted by all DEB2 plants. The response to *O. cumana* infection in sunflower lines NR5 and P96 significantly differed for *O. cumana* populations SP (race F_GV_) and GT (race G_TK_) but not for OC-94 to which the two sunflower lines showed complete resistance (Fig. 4).

**Fig. 5:**
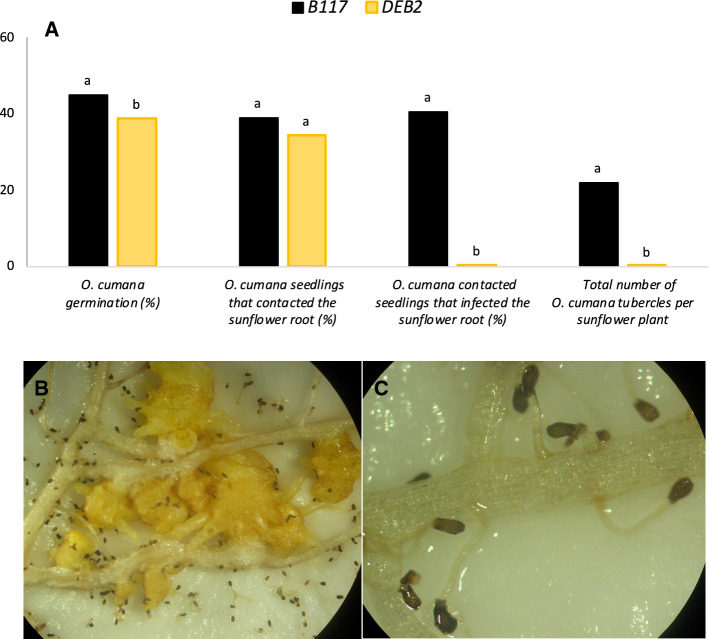
Rhizotron study of the resistant response against *O. cumana* GT (race G_TK_) of sunflower line DEB2 compared to the sunflower susceptible line B117. **a** Resistant responses observed in rhizotron. Analysis of variance was applied to replicate data. Bars with different letters are significantly different according to the Tukey test (*p* = 0.05); **b** Susceptible response with formation of *O. cumana* tubercles on B117 roots; **c** Resistant response with arrested radicle penetration in DEB2 roots

A second rhizotron assay was used to confirm the resistance of DEB2. In this assay, the resistance response of DEB2 to the most virulent *O. cumana* population GT (race G_TK_) was compared to the response of the sunflower susceptible line B117. Differences during the infection process between DEB2 and B117 were detailed by measuring: (i) the percentage of *O. cumana* seeds that germinated in the proximity of sunflower root, (ii) percentage *O. cumana* seedlings that contacted sunflower roots, (iii) percentage of sunflower-attached *O. cumana* radicles that formed tubercle and (iv) total number of *O. cumana* tubercles per sunflower plant (Fig. 5). The observed pattern of *O. cumana* GT on the resistant DEB2 and susceptible B117 was similar to that observed previously. Both sunflower lines induced high germination levels of *O. cumana* seeds, which is in accordance with the results of the germination bioassay (Fig. 3); however, B117 induced slight but significantly higher germination than DEB2. These small differences between B117 and DEB2 observed in rhizotron but not in the in vitro germination assay could be due to differences in root density developed in the rhizotron between both sunflower lines, that did not influenced the results of the in vitro germination bioassay (Fig. 3) due to the normalization made in this assay of root exudate concentration to 0.02 and 0.01 g of sunflower root/ml of root solution. After germination, *O. cumana* GT radicles were equally attracted to the roots of both sunflower lines, formed the haustoria and attached to the surface of the sunflower roots. No resistant mechanism of repellency was observed in DEB2. Differences between both sunflower lines started to be visible after *O. cumana* GT haustoria were attached to sunflower root surface. The average percentage of *O. cumana* GT haustoria that penetrated sunflower roots and formed tubercle was 40.7% in B117 roots and 0.4% in DEB2 roots. Average number of total healthy tubercles per sunflower plant formed in rhizotron was 22.0 in B117 roots and 0.17 in DEB2 roots.

#### Histological analyses

**Fig. 6:**
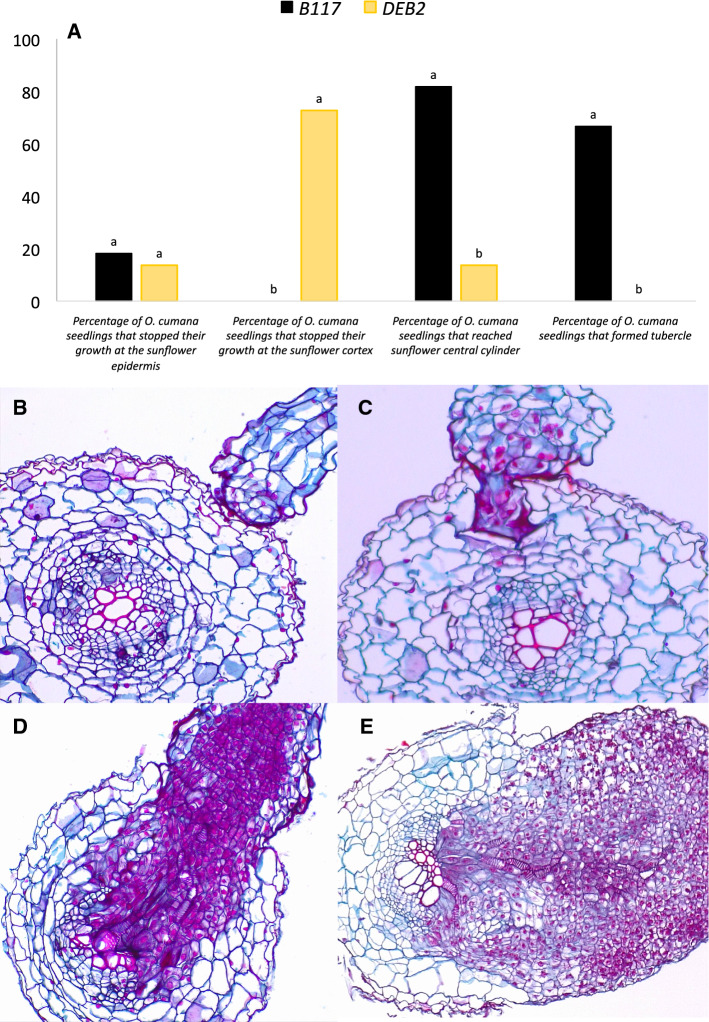
Histopathological study of the *O. cumana* GT (race G_TK_) infection process on roots of sunflower resistant line DEB2 compared to the sunflower susceptible line B117. **a** Differences during the *O. cumana* GT infection process between roots of B117 and DEB2. Analysis of variance was applied to replicate data. Bars with different letters are significantly different according to the Tukey test (*p* = 0.05); **b** *O. cumana* GT development stops at the DEB2 root epidermis; **c** *O. cumana* GT development stops at the DEB2 root cortex; **d** *O. cumana* GT development stops at the DEB2 root central cylinder; **e** *O. cumana* GT development successful infection of B117 roots with tubercle formation

Histological analyses were performed on transversal sections of infected roots of B117 and DEB2 containing attached seedlings of *O. cumana* GT population (Fig. 6). Microscopic observations of these sections revealed that root epidermis of both sunflower genotypes was equally susceptible to be penetrated by *O. cumana* GT with more than 80% of sunflower-attached radicles being able to initiate penetration into susceptible and resistant sunflower roots (Figs. 4b, 6a). In contrast, the *O. cuman*a GT seedlings differed in their ability to develop through the cortex of each sunflower line in their way to reach the sunflower central cylinder. While none of the *O. cumana* GT radicles that initiated penetration into B117 roots where stopped in the cortex, more than 70% of *O. cumana* GT attached radicles to DEB2 root were arrested at the cortex before reaching the endodermis. DEB2 cells in contact with the parasite intrusive cells presented a thickening of their walls that stained intensely red with safranin. Safranin also stained the intercellular spaces, the interface between both organisms, and inside the parasite cells (Fig. 6a, c). Only 13.6% of *O. cumana* GT radicles were able to reach the DEB2 central cylinder establishing vascular connection; however, the parasite did not develop further and stained in an intense red coloration of the *O. cumana* tissue (Fig. 6a, d). On the contrary, 81.8% *O. cumana* radicles attached to B117 root surface reached the central cylinder and established vascular connection, developing connections with host vascular tissues. By the time of sampling, 66.7% of radicles attached to B117 root surface were able to form healthy tubercle that stained in a green coloration of cytoplasm and cellulosic cell walls expected for healthy tissue, with nuclei and lignified or suberized cell walls stained red (Fig. 6a, e).

## Discussion

Resistance to *Orobanche* spp. in crop plants is generally horizontal and under polygenic, non-race specific genetic control (Pérez-Vich et al. 2013). Sunflower is a notable exception where genetic resistance to broomrape has been found in most cases to be vertical (Fernández-Martínez et al. 2015), following a gene-for-gene interaction, in which a dominant gene for host resistance interacts with a dominant avirulence gene in the parasite (Rodríguez-Ojeda et al. 2013). Such a qualitative resistance facilitates the breeding progress, since monogenic dominance is particularly well suited for F_1_ sunflower hybrid seed production (Fernández-Martínez et al. 2015). Complete monogenic dominant resistance in sunflower has been reported against all the broomrape races described so far, including races A to E (Vranceanu et al. 1980), race F (Pacureanu-Joita et al. 2004; Duriez et al. 2019), and race G (Velasco et al. 2012). A number of these major genes have been also mapped into the sunflower genome. The *Or5* gene conferring resistance to race E has been found to map to Chr3 (Tang et al. 2003; Pérez-Vich et al. 2004) and the *HaOr7* determining resistance to race F to Chr7 (Duriez et al. 2019). In this study, we have defined map position of *Or*_*Deb2*_ on Chr4 between SNP markers AX-105525205/AX-105938724 and AX-105399507, in a 0.9 cM window.

Although the monogenic dominant resistance in the DEB2 line is clearly supported by classical genetic studies conducted by Velasco et al. (2012), breeding programs aimed at introgressing the *Or*_*Deb2*_ gene into specific cultivars, and the research conducted in this study, a segregation distortion from a 1:2:1 Mendelian ratio expected for a single gene was observed in the evaluation of the F_2:3_ families of the mapping population (n = 232). Co-dominant markers mapping close to the *Or*_*Deb2*_ gene had a segregation ratio similarly distorted (Table S4), suggesting some skewed segregation of the whole genomic region in favor of alleles of the susceptible parental line. This distortion was not detected in the phenotypic analysis of the F_2_ (278 individuals) and might be due to the selection of F_2_ individuals for the mapping population with a sufficient number of F_3_ seeds for conducting the evaluations of their F_2:3_ progenies. By selecting based on the number of seeds produced, more homozygous susceptible were observed than homozygous resistant, suggesting a higher fertility of individuals with small donor region from DEB2. Since resistance in this line is coming from the wild relative *Helianthus debilis* subsp. *tardiflorus*. (Velasco et al. 2012), the data agree with observations in interspecific crosses which show that specific chromosomal segments from the wild species genome are typically reduced in frequency relative to neutral expectations (Rieseberg et al. 2000). Segregation distortion has been often observed in wide crosses (Baum et al. 1992; Faure et al. 2002), presumably reflecting the effects of competition among gametes or a selection at postzygotic stages (Zamir and Tadmor 1986), and has also been reported for other major broomrape resistance genes in sunflower originating from wild species, such as *or*_*ab-vl-8*_ (Imerovski et al. 2016).

The *Or*_*Deb2*_ was selected for resistance to the highly virulent race G (Velasco et al. 2012). However, it also confers resistance to previous less virulent races, such as race F_GV_ (Martín-Sanz et al. 2016) and race E (data not shown). This has been reported previously in sunflower for other major dominant broomrape resistance genes, in such a way that a newly discovered major dominant gene was conferring resistance to the corresponding virulent race for which it was selected, but also to previous, less virulent races (Vranceanu et al. 1980). In this study, phenotypic evaluations, and in consequence genetic mapping based on the phenotypic score, of a total of 220 F_2:3_ families tested for both race G_TK_ and race F_GV_ of broomrape yielded identical results, and no recombinants were found. This led to the question of whether the *Or*_*Deb2*_ gene is a gene of particularly strong resistance or whether it might be located in a cluster of tightly linked resistance genes, each conferring resistance to specific pathogen races. Both distinct genomic arrangements for resistance genes have been found in crop plants, in such a way that resistance genes might occur (i) as a single gene with one or more alleles encoding different resistance specificities [i.e., the flax (*Linum usitatissimum*) *L* locus, in which a single gene is found as 11 allelic variants, 10 of which encode different resistance specificities against the flax rust pathogen (Ellis et al. 1997, 1999)]; or (ii) as a series of tightly linked genes forming complex loci [i.e., the tomato (*Solanum lycopersicum*) *Cf4/9* loci (Thomas et al. 1997) or the *Rp1* locus of maize (*Zea mays*) (Collins et al. 1999; Sun et al. 2001)], in which individual genes from a cluster can confer different recognition specificities, conditioning resistance to different pathogen isolates.

Different lines of evidence point in the direction of *Or*_*Deb2*_ locus on Chr4 as being a series of tightly linked resistance genes, although further studies with larger numbers of progenies and for different races would be necessary. First, this has already been demonstrated at the molecular level in other plant-parasitic plant system in which resistance is governed by gene-for-gene interactions, such as *Striga gesnerioides* parasitizing cowpea (*Vigna unguiculata*). Li and Timko (2009) showed that when expression of the cowpea cv B301 cloned resistance gene RSG3-301, which encodes a CC (coiled coil)-NB-LRR type protein, was dampened by a virus induced gene silencing approach, susceptibility to *Striga* race 3 was restored at the time that the cultivar remained resistant to other races (2 and 5), controlled by the same locus. Second, it has been shown that the sunflower region on Chr3 in which the *O. cumana* resistance gene *Or5* was initially mapped, carries in fact different non-allelic genes controlling also resistance to *O. cumana* (Imerovski et al. 2013, 2016, 2019). Third, another major gene (*Or*_*SII*_) conferring resistance to this parasitic weed has also been mapped to sunflower Chr4 (Hassan et al. 2008; Martín-Sanz et al. 2020). This gene confers post-attachment resistance to races F and G of *O. cumana* and was mapped between the two SNP markers HT298 and HT183, which map, respectively, 6.6 and 1 cM distal and proximal to *Or*_*SII*_ (Hassan et al. 2008), and between non-published proprietary markers (Martín-Sanz et al. 2020). The HT298-HT183 marker interval is located 2.8 cM upstream of the RGC marker RCG127 (Radwan et al. 2008), which in turns maps 8.8 cM distal to *Or*_*Deb2*_ (Fig. 1, this study). On the other hand, blast searches of the HT183 context sequence (Hassan et al. 2008) against the sunflower XRQr2.0 sunflower genome assembly show this marker to be physically located at 7.643 Mbp (BLAST E-value 9.96 × 10^–144^). The distal markers flanking *Or*_*Deb2*_ AX-105938724 and AX-105525205 are located at physical positions 7.880 Mbp and 7.892 Mbp, respectively (Table S4), and *Or*_*Deb2*_ maps 0.7 cM downstream them (Fig. 1). Comparison of map and physical positions of markers flanking *Or*_*SII*_ and *Or*_*Deb2*_ suggest in consequence that these genes might be non-allelic and located at tightly linked positions in Chr4, although this should be confirmed through fine mapping and allelic crosses. Their non-allelic nature is supported by the completely different nature of the *Or*_*SII*_ and *Or*_*Deb2*_ associated resistance mechanisms, as it will be discussed below, and their different origin, with *Or*_*Deb2*_ directly transferred from wild sunflower species *Helianthus debilis* subsp. *tardiflorus* (Velasco et al. 2012) and *Or*_*SII*_ identified within a private breeding program in the course of the evaluation of a proprietary sunflower germplasm collection (Hassan 2003). Finally, a cluster of putative candidate resistance genes has been identified in the genomic region in which *Or*_*Deb2*_ has been located, as it will be discussed below, thus supporting that the *Or*_*Deb2*_ locus might actually be a cluster of genes conferring different *O. cumana* recognition specificities.

In addition to *Or*_*Deb2*_ and *Or*_*SII*_, the upper segment of Chr4 has also been reported to harbor other sunflower major dominant genes conferring resistance to pathogens other than *O. cumana* (Qi et al. 2015; Zhang et al. 2017; Pecrix et al. 2018; Liu et al. 2019; Ma et al. 2019). Clustering of genes conferring resistance to different pathogens has already been described in sunflower, as for example the complex mixed clusters on the lower end of Chr13 which harbor the downy mildew [caused by the oomycete *Plasmopara halstedii* (Farl.) Berlese & de Toni] *Pl*_*5*_, *Pl*_*8*_, *Pl*_*21*_, and *Pl*_*22*_, and the rust (caused by the fungus *Puccinia helianthi* Schw.) *R*_*4*_, *R*_*13a*_, *R*_*13b*_, *R*_*16*_, and *R*_*Adv*_ resistance genes (Bachlava et al. 2011; Vincourt et al. 2012; Pecrix et al. 2018; Liu et al. 2019), as well as in other crops where it is a well-documented phenomenon (Hulbert et al. 2001). So far, the *Pl*_*17*_, *Pl*_*19*_* Pl*_*27*_–*Pl*_*29*_ and *Pl*_*33*_ genes for resistance to downy mildew have been located in the upper segment of Chr4 (Qi et al. 2015; Zhang et al. 2017; Pecrix et al. 2018; Liu et al. 2019; Ma et al. 2019). Molecular dissection of this Chr4 downy mildew resistance gene cluster has precisely mapped *Pl*_*17*_, *Pl*_*19*_ and *Pl*_*33*_ at positions from 5.69 to 5.71 Mbp, 6.68 to 6.71 Mbp, and 4,21 to 5,77 Mbp, respectively, of the HanXRQr1.0 assembly (Ma et al. 2019; Lui et al. 2019), while the flanking markers place *Pl*_*27*_ in an interval between 2.18 and 6.40 Mbp, *Pl*_*28*_ between 6.62 and 8.42 Mbp, and *Pl*_*29*_ between 6.93 and 7.07 Mbp in the HanXRQr1.0 assembly (Pecrix et al. 2018). Therefore, the physical interval in which the Chr4 *Pl* genes have been located does not overlap with that for *Or*_*Deb2*_ (using positions in the HanXRQr1.0 assembly), with the *Pl* genes located upstream of *Or*_*Deb2*_.

Following models from other plant species in which resistance is governed by major race-specific dominant genes, resistance to broomrape was hypothesized to be conferred by intracellular receptors that contain a predicted NB-LRR structure (Lu et al. 2000; Imerovski et al. 2019), as it has also been reported for other plant-parasitic plant interactions (Li and Timko, 2009). This was supported by the existence of clusters of recognition-dependent disease resistance genes which encode NB-LRR proteins on the upper half of chromosomes 3 and 4 (Radwan et al. 2008), where *Or* genes have been mapped. However, the mapped NB-LRR loci in the present study (Iasnip SNP markers; Fig. 4) were not tightly linked to *Or*_*Deb2*_. In fact, their physical position (Table S4) was more coincident to that of the Chr4 physically mapped downy mildew resistance genes (Pecrix et al. 2018). In addition, the unique gene conferring resistance to *O. cumana* cloned to date (*Or7*) encodes a LRR receptor-like kinase (Duriez et al. 2019). These data pointed to the fact that *Or*_*Deb2*_ resistance gene was not from the NB-LRR protein family. This has been confirmed in the present study, since none of the genes lying in the *Or*_*Deb2*_-1.38 Mbp interval was from the intracellular NB-LRR receptors class.

Gene identification and organization within the 1.38 Mbp region in which *Or*_*Deb2*_ has been mapped shows that this gene is located in a cluster of genes encoding RLKs and RLPs. Their abundance in this region, the fact that the only cloned *O. cumana* resistance gene is a RLK (Duriez et al. 2019), and the essential role of these kind of genes in different biotic stress responses in plants (Fritz-Laylin et al. 2005; Kruijt et al. 2005; Wang et al. 2008; Lehti-Shiu et al. 2009; Dievart et al. 2020) pointed the nine RLK and RLP genes identified as being the best candidates for *Or*_*Deb2*_. These nine genes belong to two highly structurally different groups. One group are RLKs characterized for carrying one or two kinase domains, with serine/threonine kinase specificity, no LRR or extracellular domains, and four of them classified within the RLK/Pelle CrRLK1L-1-like subfamily. The inclusion in this subfamily was due to the homology found in the kinase domain, since this subfamily is characterized also by having malectin-like extracellular ligand binding and transmembrane domains (Diervart et al. 2020), which were not present in the RLKs identified in this study. The other group are RLPs which have a large LRR extracellular domain. In two of these genes, small transmembrane and cytoplasmic domains were also identified. Unlike RLKs, these RLPs lack any obvious domain for intracellular signaling.

The genomic localization and sequence similarity of the RLK and RLP genes in the *Or*_*Deb2*_-1.38 Mbp suggest their arrangement in clusters originated, at least in part, from tandem duplications. This organization of RLP and RLK genes clustering together, as well as their distribution in tandem repeats, has already been shown in Arabidopsis (Shiu and Bleeker 2001, 2003) and in crops such as rice (Zhang et al. 2005). This has been suggested to be a mechanism for the expansion of this gene family and for providing opportunities to acquire new gene functions or specificities (Shiu and Bleeker 2003). Interestingly, PANTHER family classification grouped the *Or*_*Deb2*_-1.38 Mbp (boundaries from 7.9 to 9.3 Mbp in the XRQr2.0 assembly) RLKs and RLPs into subfamilies of 26 and 17 sunflower homologs, respectively, which in both cases were mostly (65.4% of the RLKs and 82.4% of the RLPs) located tightly close to this interval on Chr4, with the majority of the RLKs (12 of them) located from 6.5 to 7.4 Mbp, and of the RLPs (10 of them) from 9.3 to 10.8 Mbp. These results might suggest that the *Or*_*Deb2*_-1.38 Mbp sequence is within a region spanning at least 4.3 Mbp, from physical positions 6.5 to 10.8 Mbp, that probably contains different clusters of these specific RLKs and RLPs.

The putative gene candidates identified in the *Or*_*Deb2*_-1.38 Mbp interval raised the question if any of them may be the causal gene conferring resistance to *O. cumana*. On the one hand, there exist structural similarities of the RLPs and RLKs detected in this interval with other already described resistance genes. Disease resistance genes coding for RLPs with a large extracellular LRR domain and a short cytoplasmic tail that lacks motifs for intracellular signaling include the tomato *Cf* and *Ve* genes that provide resistance against the leaf mold fungus *Cladosporium fulvum* and vascular wilt pathogens of the genus *Verticillium*, respectively (Jones et al. 1994; Kawchuk et al. 2001; Fradin et al. 2011), the apple (*Malus domestica*) *HcrVf* genes that confer resistance to the scab fungus *Venturia inaequalis* (Malnoy et al. 2008), and the *Brassica napus LepR3* and *Rlm2* genes that render race-specific resistance to the fungal pathogen *Leptosphaeria maculans* (Larkan et al. 2013; Larkan and Borhan 2015). Also, an RLP-based recognition of the parasitic plant *Cuscuta reflexa* has been described in tomato (Hegenauer et al. 2016). In addition, RLKs lacking extracellular and transmembrane domains have also been associated to disease resistance. In our study, we identified two types, one having one kinase domain and other with two fused kinase domains in which only one of them (that with higher homology to Arabidopsis RLK genes) showed a serine/threonine active site and a ATP binding site. Kinase-only disease resistance genes include for example the tomato *Pto* gene determining resistance to the plant pathogenic bacterium *Pseudomonas syringae* pv *tomato*, which sense intracellular effectors and activate NB-LRR mediated immunity (Ntoukakis et al. 2014). In the case of the dual-kinase genes identified in this study, they show structural similarities to tandem kinase-pseudokinase genes underlying the barley *Rpg1* (Brueggeman et al. 2002) and the wheat *Yr15* (Klymiuk et al. 2018) and *Sr60* (Cheng et al. 2020) genes which determine resistance to rust fungi diseases caused by *Puccinia* spp. On the other hand, and since it has been shown that resistance gene repertories vary at phylogenetic scales within genera and species (Steinbrenner 2020), it might also be hypothesized that none of the already identified genes coding for RLKs or RLPs in the XRQ assembly might represent an allele of the functional *Or*_*Deb2*_ gene, which has been introgressed from the wild sunflower species *Helianthus debilis* subsp*. tardiflorus* (Velasco et al. 2012). This has been shown for example with the RLP ELR (elicitin response) which confers enhanced resistance to the oomycete pathogen *Phytophthora infestans* in potato (*Solanum tuberosum*). This gene was identified based on genus-level variation within Solanaceae in the wild potato *Solanum microdontum*, but it was absent in the reference genome of potato (Du et al. 2015). Also, it has been suggested that the expansion of clustered RLKs and RLPs involved in resistance and defense responses may be related to localized gene duplications, which determine gene rearrangements and therefore the possible acquisition of new functionalities and novel ways to recognize extracellular signals such as pathogen effectors (Shiu et al. 2004; Zhang et al. 2005). For example, Xa21D, an RLP resembling the extracellular domains of RLKs and in close genomic proximity to a related RLK, illustrates this. Xa21D presumably is derived from a duplication event that gave rise to this gene and *Xa21*, a rice RLK with both LRR-extracellular and kinase-intracellular domains conferring resistance to the bacterial pathogen *Xanthomonas oryzae* pv. *oryzae* (Wang et al. 1998), which shares similarity with the recently cloned sunflower *HaOr7* gene (Duriez et al. 2019). Subsequent transposon insertion in Xa21D resulted in a truncated protein with only the extracellular domain, which confers partial disease resistance (Wang et al. 1998). This might underlie new functionalities in the resistant line DEB2. In any case, it would be expected that the putative allele, if present in the genome assembly of the XRQ *O. cumana* susceptible line, may carry mutations determining a non-functional protein, such as stop codons or insertions/deletions, already described in reference genomes carrying the susceptible alleles from cloned resistance genes (Duriez et al. 2019; Chen et al. 2020). Finally, local misassembles and annotation errors cannot be completely ruled out, although it has been found in this study much more consistency between the reference genome assembly XRQ2.0 and the location of markers than in the previous assembly XRQ1.0. Further *Or*_*Deb2*_ fine mapping and the genome sequence of the resistant line DEB2 would be necessary to discern among the different hypotheses regarding the nature of the functional *Or*_*Deb2*_ gene.

The use of resistant sunflower cultivars is compromised by the emergence of more virulent races of broomrape. Because a range of host resistance mechanisms have been identified, each blocking different infection stages, pyramiding genes each blocking successive steps in the infection process will increase the durability of sunflower resistance (Fernández-Aparicio et al. 2020). In sunflower, few studies have characterized which *O. cumana* developmental stages are affected by resistance responses induced by single-inherited dominant *Or* genes. Early studies by Antonova and Ter Borg (1996) showed that interaction between peroxidases from *O. cumana* and the sunflower lignin precursors was the basis of the resistance of cultivars with the *Or3* gene, in such a way that the extracellular peroxidase in *O. cumana* race C reacts with phenolic compounds, which are lignin precursors of the host, resulting in host resistance due to the formation of lignin layers in sunflowers possessing the *Or3*. This gene was proposed to control this accumulation of phenolic compounds and lignin formation. In contrast, the absence of extracellular peroxidase in *O. cumana* race D prevented lignin formation and enabled the parasite to attach to the host vascular system, overcoming *Or3* resistance. More recently, the *O. cumana* developmental stages affected by the resistance genes *HaOr7*, conferring resistance to race F, and *Or*_*SII*_, determining resistance to races F and G have been reported. *HaOr7* prevents *O. cumana* from connecting to the vascular system of sunflower roots owing to an incompatible attachment (Duriez et al. 2019), while *Or*_*SII*_ determines a more delayed activation of resistance responses, occurring after vascular connections between the parasite and the host have been established (Martín-Sanz et al. 2020). Martín-Sanz et al. (2020) also described the necrosis of parasite structures after tubercle development, probably due to toxicity of the host released phenolic compounds at the infection point. Results from rhizotron and histological analyses carried out in this study show that *Or*_*Deb2*_ determines resistance to *O. cumana* infection through a post-attachment defensive mechanism that, contrarily to the compatible interactions established by *O. cumana* populations OC-94, SP, and GT (races E_GV_, F_GV_ and G_TK_ respectively) with roots of the susceptible line B117, blocks the radicles ability of all *O. cumana* races tested from developing tubercles, that is before developing connections with host vascular tissues, which is more similar to the *Or7* resistance mechanism. Histological sections showed that DEB2 sunflower line had minimal number of *O. cumana* race G haustoria penetrating the endodermis, being the parasite mainly stopped at the cortex by means of an encapsulation layer. The cortex has been reported to be a major barrier to *O. cumana* infection in resistant sunflower cultivars (Dörr et al. 1994; Echevarría-Zomeño et al. 2006), as well as in the wild *Helianthus* species *H. debilis* subsp. *debilis* (Labrousse et al. 2001). More specifically, Dörr et al. (1994) and Labrousse et al (2001) reported that resistant sunflower prevents the penetration of *O. cumana* haustorium with the formation of an isolation layer in the cortex around the parasite tissue, while Echevarria-Zomeño et al. (2006) indicated that resistance response against *O. cumana* in the cortex is created by thickening and reinforcement by suberization and protein cross-linking of host cell walls at the host-parasite interface and by sunflower excretion of phenolic compounds creating a toxic environment at the infection site.

Seeds of *Orobanche* and *Phelipanche* species germinate when they detect germination stimulants released from host roots, mainly strigolactones (Xie et al. 2010). A notable exception is the interaction of sunflower-*O. cumana*. Although sunflower roots exudate strigolactones such as orobanchyl acetate and 5-deoxystrigol (Yoneyama et al. 2011) and *O. cumana* seed germination can be induced by strigolactones (Fernández-Aparicio et al. 2011), the specificity of *O. cumana* for sunflower infection has been in part related to their higher sensitivity to non-strigolactone compounds such as dehydrocostus lactone and other sesquiterpene lactones exuded by sunflower (Joel et al. 2011) that are specifically active at low concentrations on *O. cumana* but not in other broomrape species (Joel et al. 2011; Cala et al. 2017). Although low germination-inducing varieties can be resistant to broomrape infection, and their identification is an obvious target for breeding (Yoder and Scholes 2010), results from this study indicated that resistance conferred by *Or*_*Deb2*_ is not related to low induction of *O. cumana* germination, since germination of *O. cumana* seeds was similarly stimulated by exudates of the resistant line DEB2 and the rest of the sunflower lines tested, including the susceptible line B117. These results agree with those reported for the resistance genes *Or3*, *Or7*, and *Or*_*SII*_ (Antonova and Ter Borg 1996; Duriez et al. 2019; Martín-Sanz et al. 2020), and also with those indicated for the proprietary race F resistant genotype HE-399999 (Echevarria-Zomeño et al. 2006), whose genetics has not been reported. Our study has however detected varietal differences for germination induction of *P. ramosa*, a broomrape species with a broad host range that can infect sunflower (Parker et al. 2013) and whose seeds do not germinate with low concentrations of sunflower-specific sesquiterpene lactones (Cala et al. 2017) as it occurs in other *Phelipanche* species (Joel et al. 2011). We have found higher levels of *P. ramosa* germination induced by root exudates of sunflower line NR5 in comparison with the rest of sunflower lines tested, which could be explained by either (i) higher NR5 exudation of sesquiterpene lactones that could stimulate higher levels of *P. ramosa* germination or (ii) similar exudation among sunflower varieties sesquiterpene lactones but increased NR5 exudation of strigolactones more active in *Phelipanche* species than in *O. cumana*. Both hypotheses could be discarded according to the recent work published by Rial et al. (2021) which studied the concentration of sesquiterpene lactones and strigolactones exuded by roots of sunflower lines NR5, B117 and P96 also used in this research. Their results indicated that the concentration of sesquiterpene lactones (dehydrocostus lactone and costunolide) and strigolactones (orobanchyl acetate) was either similar or lower in NR5 when compared with B117 and P96 (Rial et al. 2021). Varietal differences in exudation of either uncharacterized germination stimulants or germination inhibitors specifically active on *P. ramosa* or synergistic effects among exuded molecules could be responsible for the NR5 increased *P. ramosa* germination. Sunflower is known to synthesize compounds with allelopathic activity (Macías et al. 1998) that could be acting against *P. ramosa* germination but no information exists on differences in concentrations of germination inhibitors among the sunflower cultivars studied in our work.

In conclusion, our study shows that the major gene *Or*_*Deb2*_ determines a post-attachment resistance response that blocks *O. cumana* development mainly at the cortex before the establishment of host-parasite vascular connections, and it is located within a 1.38 Mbp interval on sunflower Chr4. This region has a cluster containing nine receptor-like proteins lacking a cytoplasmic kinase domain and receptor-like kinases lacking an extracellular LRR domain, which due to their involvement in disease resistance in other plants, and also in resistance to *O. cumana* in sunflower, are valuable candidates for *Or*_*Deb2*_. Although further investigation is necessary to test if any of the identified RLPs/RLKs is the causal gene for resistance to *O. cumana*, these results offer an interesting opportunity to gain a deeper understanding of the genomic organization, genetic function and evolution of this kind of clusters in the sunflower genome. *Or*_*Deb2*_ has been demonstrated to confer resistance not only to race G from Turkey by also to several other races G from Eastern Europe countries, Ukraine, Russia and Spain (Martín-Sanz et al. 2016). Increased knowledge about its interaction with *O. cumana* will contribute to build more durable and sustainable breeding strategies based on genetic resistance and has direct application to sunflower breeding programs for the marker-assisted introgression of *O. cumana* resistance genes into elite sunflower germplasm and to identify *Or*_*Deb2*_ by a map-based cloning strategy.

## Supplementary Information

Below is the link to the electronic supplementary material.

Supplementary file1 (PDF 515 kb)

Supplementary file2 (XLS 42 kb)

Supplementary file3 (XLSX 13 kb)

Supplementary file4 (XLSX 12 kb)

Supplementary file5 (XLSX 14 kb)

Supplementary file6 (XLSX 16 kb)
