# Supplementary material for: Genetic and physiological characterization of sunflower resistance provided by the wild-derived OrDeb2 gene against highly virulent races of Orobanche cumana Wallr
Source: Theor Appl Genet. 2021 Nov 6;135(2):501–25. doi: 10.1007/s00122-021-03979-9 (PMC8866362; doi:10.1007/s00122-021-03979-9)
Supplement: Supplementary file 1 — Supplementary file1 (PDF 515 kb) [file 122_2021_3979_MOESM1_ESM.pdf]

Fig S1. Examples of InterPro domain constitution and TAIR BlastP analyses of kinase loci in the Or_{Deb2}-1,38 Mbp region

1- Locus Chr04g0142291 (XP_022033748.1, 662 aa). Putative protein kinase RLK-Pelle-CrRLK1L-1 family

XP_022033748.1 InterPro domain constitution

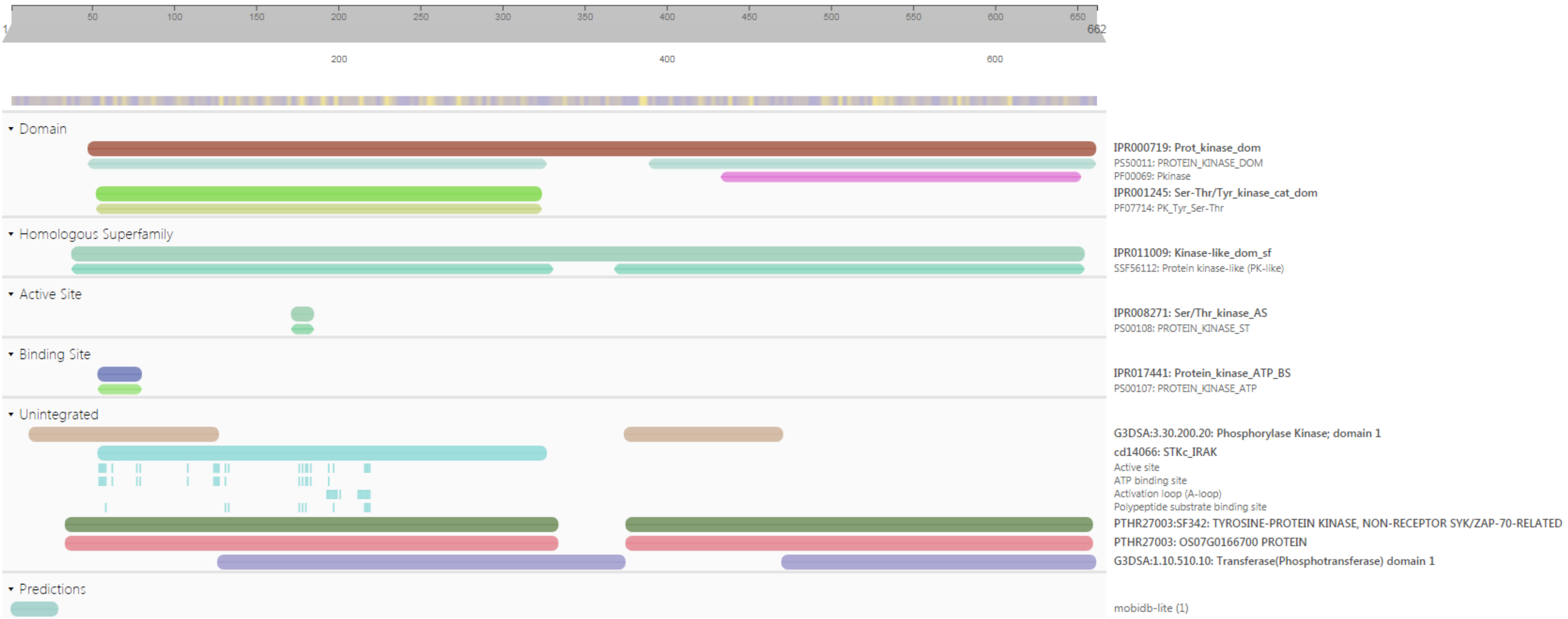

XP_022033748.1 TAIR BlastP results (1 e-values lower than 1×10^{-70})

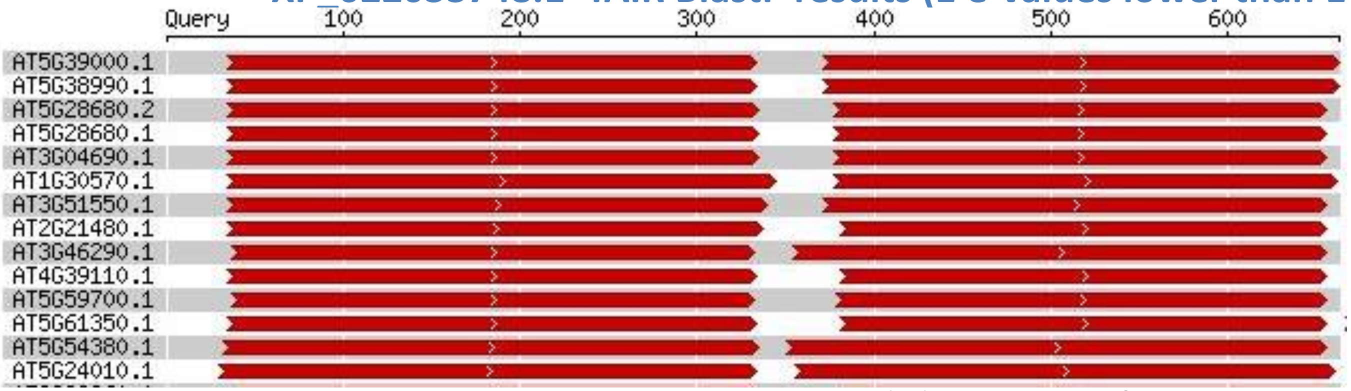

Left kinase region of XP_022033748.1:
Identity to kinase region of Atg39000.1: 47%
Similarity to kinase region of Atg39000.1: 65%

Right kinase region of XP_022033748.1 :
Identity to kinase region of Atg39000.1: 31%
Similarity to kinase region of Atg39000.1: 50%

2.- Locus Chr04g0142321 (XP_022031817.1). Putative protein kinase RLK-Pelle-CrRLK1L-1 family

XP_022033817.1 InterPro domain constitution

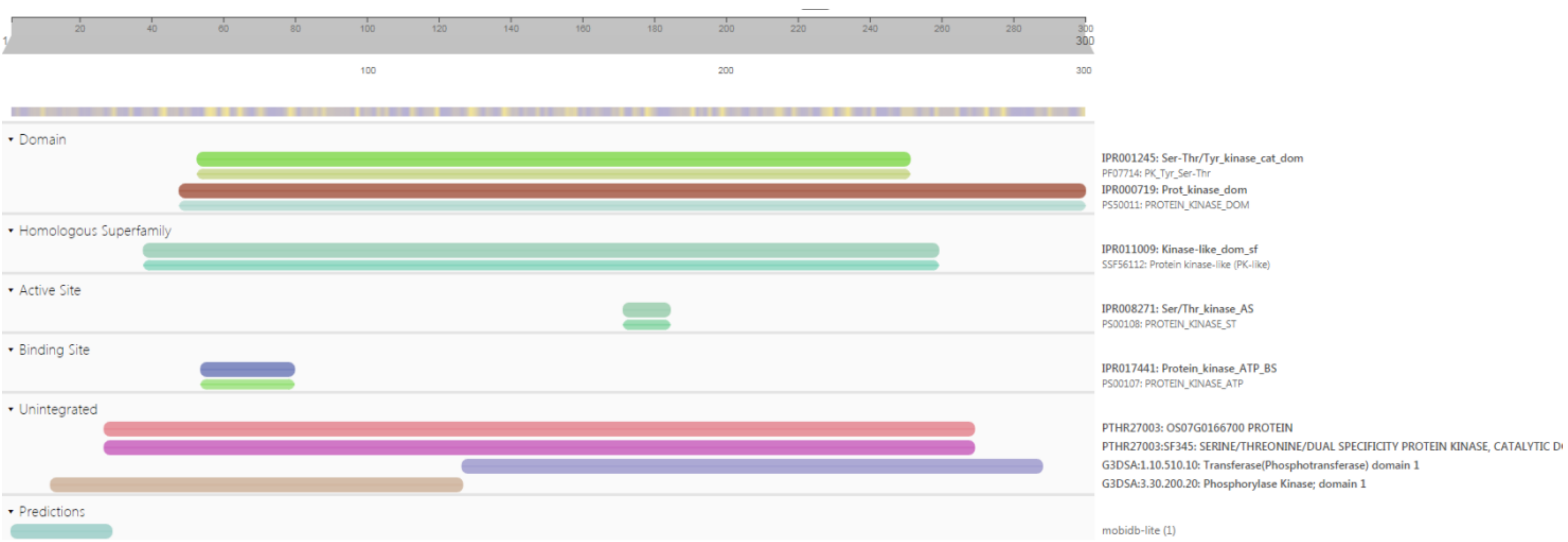

XP_022033817.1 TAIR BlastP results (1 e-values lower than 1×10^{-70})

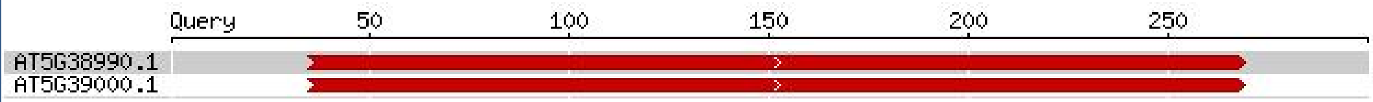

Identity to kinase region of Atg38990.1: 51%

Similarity to kinase region of Atg38990.1: 67%

3.- Locus Chr04g0142271 (XP_022031819.1) Putative non-specific serine/threonine protein kinase

XP_022033819.1 InterPro domain constitution

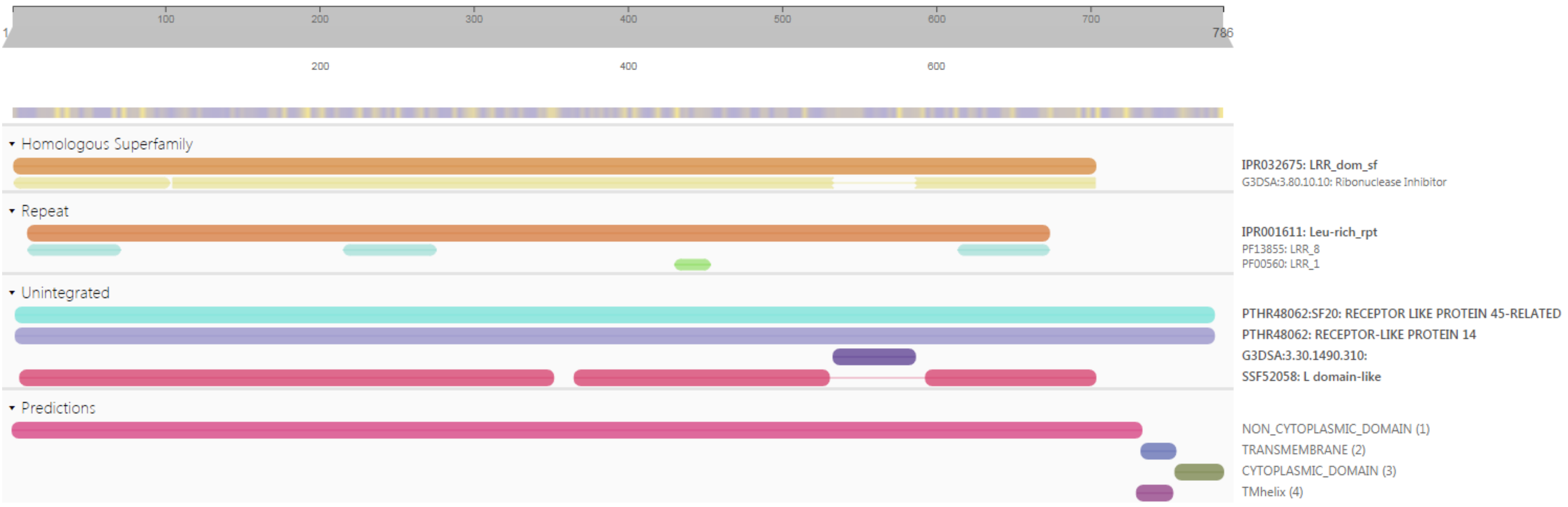

XP_022033817.1 TAIR BlastP results (1 e-values lower than 1×10^{-130})

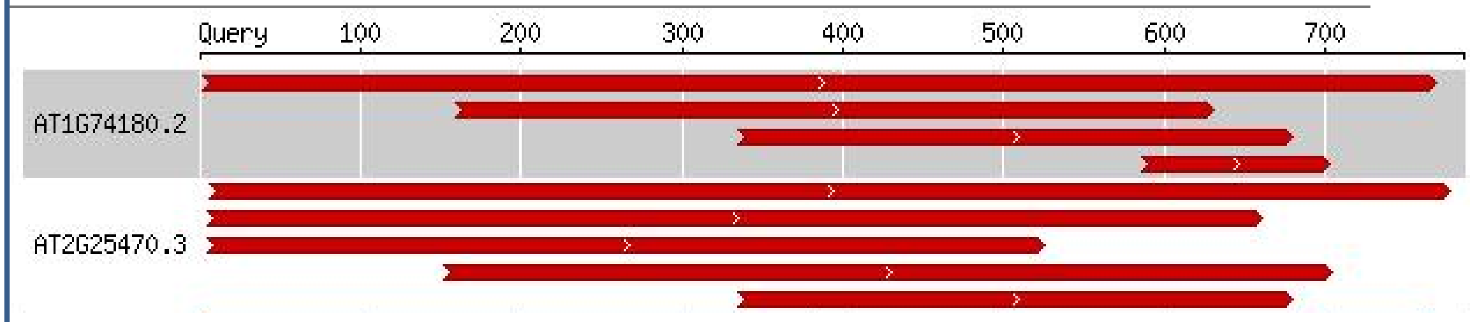

Identity to Atg1g74180 (RLP14): 39%

Similarity to Atg1g74180 (RLP14): :56%
